# Phosphorylation Modulates Clearance of Alpha-Synuclein Inclusions in a Yeast Model of Parkinson's Disease

**DOI:** 10.1371/journal.pgen.1004302

**Published:** 2014-05-08

**Authors:** Sandra Tenreiro, Madalena M. Reimão-Pinto, Pedro Antas, José Rino, Donata Wawrzycka, Diana Macedo, Rita Rosado-Ramos, Triana Amen, Meytal Waiss, Filipa Magalhães, Andreia Gomes, Cláudia N. Santos, Daniel Kaganovich, Tiago Fleming Outeiro

**Affiliations:** 1 Instituto de Medicina Molecular, Faculdade de Medicina da Universidade de Lisboa, Lisboa, Portugal; 2 Department of Genetics and Cell Physiology, Institute of Experimental Biology, University of Wroclaw, Wroclaw, Poland; 3 Instituto de Tecnologia Química e Biológica, Universidade Nova de Lisboa, Oeiras, Portugal; 4 Department of Cell and Developmental Biology, Alexander Silberman Institute of Life Sciences, Hebrew University of Jerusalem, Jerusalem, Israel; 5 Instituto de Biologia Experimental e Tecnológica, Oeiras, Portugal; 6 Instituto de Fisiologia, Faculdade de Medicina da Universidade de Lisboa, Lisboa, Portugal; 7 Department of NeuroDegeneration and Restorative Research, Center for Nanoscale Microscopy and Molecular Physiology of the Brain, University Medical Center Göttingen, Göttingen, Germany; University of Pennsylvania, United States of America

## Abstract

Alpha-synuclein (aSyn) is the main component of proteinaceous inclusions known as Lewy bodies (LBs), the typical pathological hallmark of Parkinson's disease (PD) and other synucleinopathies. Although aSyn is phosphorylated at low levels under physiological conditions, it is estimated that ∼90% of aSyn in LBs is phosphorylated at S129 (pS129). Nevertheless, the significance of pS129 in the biology of aSyn and in PD pathogenesis is still controversial. Here, we harnessed the power of budding yeast in order to assess the implications of phosphorylation on aSyn cytotoxicity, aggregation and sub-cellular distribution. We found that aSyn is phosphorylated on S129 by endogenous kinases. Interestingly, phosphorylation reduced aSyn toxicity and the percentage of cells with cytosolic inclusions, in comparison to cells expressing mutant forms of aSyn (S129A or S129G) that mimic the unphosphorylated form of aSyn. Using high-resolution 4D imaging and fluorescence recovery after photobleaching (FRAP) in live cells, we compared the dynamics of WT and S129A mutant aSyn. While WT aSyn inclusions were very homogeneous, inclusions formed by S129A aSyn were larger and showed FRAP heterogeneity. Upon blockade of aSyn expression, cells were able to clear the inclusions formed by WT aSyn. However, this process was much slower for the inclusions formed by S129A aSyn. Interestingly, whereas the accumulation of WT aSyn led to a marked induction of autophagy, cells expressing the S129A mutant failed to activate this protein quality control pathway. The finding that the phosphorylation state of aSyn on S129 can alter the ability of cells to clear aSyn inclusions provides important insight into the role that this posttranslational modification may have in the pathogenesis of PD and other synucleinopathies, opening novel avenues for investigating the molecular basis of these disorders and for the development of therapeutic strategies.

## Author Summary

Protein aggregation is a common hallmark in neurodegenerative disorders, but is also associated with phenotypic plasticity in a variety of organisms, including yeasts. Alpha-synuclein (aSyn) forms aggregates that are typical of synucleinopathies, and is phosphorylated at S129, but the significance of phosphorylation in the biology and pathophysiology of the protein is still controversial. Exploring the power of budding yeast, we found phosphorylation reduced aSyn toxicity and inclusion formation. While inclusions formed by WT aSyn were homogeneous, those formed by S129A aSyn were larger and heterogeneous. Interestingly, clearance of aSyn inclusions was reduced in cells expressing S129A aSyn, correlating with deficient autophagy activation. The finding that phosphorylation alters the ability of cells to clear aSyn inclusions provides novel insight into the role phosphorylation may have in synucleinopathies, and suggests posttranslational modifications might constitute switches cells use to control the aggregation and clearance of key proteins, opening novel avenues for the development of therapeutic strategies for these devastating disorders.

## Introduction

Protein misfolding and aggregation is an unavoidable and widespread problem in biology. Cells evolved a series of quality control mechanisms to ensure overall proteostasis and, in some cases, to exploit the plasticity of diverse conformational states, including those concealed in protein aggregates, as in the case of certain types of prions. In other instances, protein aggregates can be detrimental [1], [2]. Protein inclusions made of alpha-Synuclein (aSyn), known as Lewy bodies (LBs) are the pathological hallmark of Parkinson's disease (PD) and other disorders known as synucleinopathies [3], [4]. The normal function of aSyn is still unclear, but it is thought to be involved in the regulation of dopamine neurotransmission, vesicular trafficking and in synaptic function and plasticity [5]. Although aSyn is phosphorylated at low levels under physiological conditions, a striking 90% of aSyn is phosphorylated at S129 (pS129) in LBs [6]. However, the significance of pS129 in the pathogenesis of synucleinopathies is unresolved. While studies in *Drosophila melanogaster* [7] and transgenic mouse models of PD [8] showed that pS129 aSyn was pathogenic, studies in rats and in *Caenorhabditis elegans* failed to associate toxicity with phosphorylation and suggested a role of pS129 in the attenuation of aSyn induced neuronal dysfunction [9], [10]. However, no differences in toxicity or aggregate formation were seen in a rat model [11]. Whether pS129 promotes or prevents aggregation remains largely controversial [12]–[14].

The yeast *Saccharomyces cerevisiae* is a powerful model for the study of protein misfolding due to the high conservation of the quality control systems with all other eukaryotes, including humans [15]. Although *S. cerevisiae* lacks an aSyn ortholog, heterologous expression of the protein induces toxicity in a concentration dependent manner and is associated with the formation of cytoplasmic protein inclusions [16]. Moreover, a network of highly conserved aSyn interactors was identified, suggesting the protein can be studied using simple models such as yeast, worms, or flies, in addition to mammalian models [17], [18]. Several pathways involved in aSyn-associated toxicity in yeast are conserved in other eukaryotic models of PD. This is the case of apoptosis [16], lipid droplet accumulation [16], mitochondrial dysfunction [19], [20], proteasome impairment [16], [21], [22], oxidative stress [21], [23], autophagy and mitophagy dysfunction [24], [25], vesicle trafficking defects [16], [26], and ER-to-Golgi trafficking impairment [20], [27], [28].

PD pathogenesis is thought to be exacerbated from inefficient protein clearance as consequence of dysfunction in protein degradation [29], [30]. Clearance of aSyn can occur through direct proteolysis [31], the ubiquitin-proteasome system (UPS) [32] and/or chaperone-mediated autophagy (CMA) [33]. However, under pathological conditions, aSyn inhibits the proteasome [22], [34] and impairs chaperone-mediated autophagy, leading to the upregulation of macroautophagy (hereafter referred as autophagy) [35], [36]. Autophagy dysfunction plays a central role in PD [24], [35], [37] and was shown to be required for aSyn degradation under pathological conditions [38]. Accordingly, increasing evidence suggests the existence of a complex cross-talk between different forms of autophagy and also between autophagy and the proteasomal degradation pathway, processes known to play distinct roles in the clearance of specific species of aggregated aSyn [22], [34], [35], [39], [40].

In yeast, the clearance of aSyn inclusions has been associated with both UPS and autophagocytic degradation pathways [41]. However, recent observations suggest the UPS may be less relevant in mediating aSyn clearance [25].

Here, we explored the power of yeast genetics in order to gain mechanistic insights into the role of S129 phosphorylation on aSyn biology. Using mutants of aSyn that attempt to mimic either phosphorylated (S129E) or unphosphorylated (S129A or S129G) states aSyn, we found that blocking phosphorylation increases aSyn toxicity and promotes the formation of cytosolic inclusions. Our data are consistent with the involvement of phosphorylation in the clearance of aSyn via autophagy.

Altogether, our study provides insight into the role of S129 aSyn phosphorylation and opens novel avenues for additional studies in higher model systems.

## Results

### Blockade of aSyn S129 phosphorylation promotes toxicity and inclusion formation

**Figure 1:**
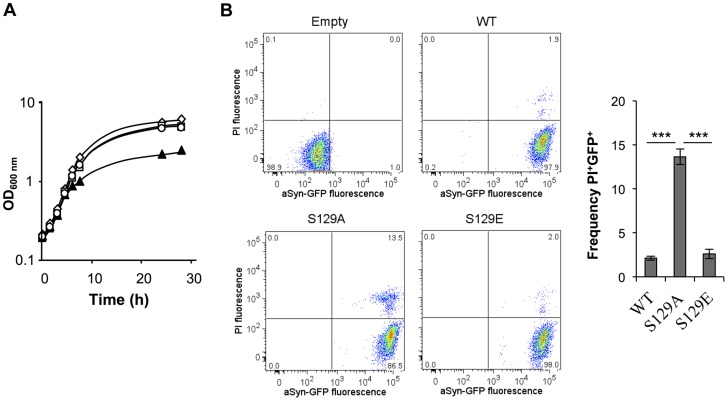
S129A aSyn is more toxic for yeast cells than the WT aSyn. (A) Growth curve based on culture OD_600nm_ of yeast cells expressing WT (□), S129A (▴) or S129E (○) aSyn-GFP, compared to cells that are not expressing the human protein (◊). Cells used as inoculum were exponential-phase cells cultivated in raffinose medium that at time zero were transferred to galactose medium to induce aSyn-GFP expression. (B) aSyn-GFP versus Propidium Iodide (PI) fluorescence and Frequency of PI and GFP positive cells assessed by flow cytometry, in the indicated yeast cells, after 6 hours of aSyn expression induction (***p<0.001; one way ANOVA with Bonferroni's multiple comparison test). A representative result is shown from at least five independent experiments. Values represent the mean ± SD.

To investigate the effect of aSyn phosphorylation in yeast, we used strains carrying two copies of human *SNCA* cDNA integrated in the genome, encoding either wild-type (WT), S129A, S129G, or S129E mutant aSyn fused to GFP (aSyn-GFP), in order to block (S129A or S129G) or mimic (S129E) phosphorylation, under the regulation of a galactose inducible promoter (*GAL1*) (Table 1). These strains were previously described and characterized [42] and display a moderate level of aSyn toxicity compared to strains described in other studies [16], [27], [28]. Growth and viability (colony-forming units, CFUs) of the different strains were assessed upon induction of aSyn expression (Figure 1A and S1A). We observed an initial lag phase of ∼2 hours in cells expressing either WT or S129E aSyn-GFP, compatible with the carbon source switch (Figure S1A). Subsequently, these strains resumed growth and behaved similarly, growing slightly slower that the control cells (Figure 1A).

**Table 1:** Strains used in this study.

	Description	Reference
W303-1A	*MAT*a; *can1-100*; *his3-11,15*; *leu2-3,112*; *trp1-1*; *ura3-1*; *ade2-1*	[95]
W303-1B	*MAT*α; *can1-100*;*his3-11, 15*; *leu2-3*, *112*; *trp1-1*; *ura3-1*; *ade2-1*	[95]
VSY71	W303-1A *trp1-1::pRS304 TRP1+*; *ura3-1*:: *pRS306 URA3+*	[96]
VSY72	W303-1A *trp1-1:: pRS304 GAL1pr*-*SNCA*(WT)-*GFP TRP1+*; *ura3-1*:: *pRS306 pRS306GAL1pr*-*SNCA*(WT)-*GFP::URA3+*	[96]
VSY73	W303-1A *trp1-1:: pRS304 GAL1pr*-*SNCA*(S129A)-*GFP TRP1+*; *ura3-1*:: *pRS306 GAL1pr*-*SNCA*(S129A)-*GFP::URA3+*	[96]
VSY74	W303-1A *trp1-1:: pRS304 GAL1pr*-*SNCA*(S129E)-*GFP TRP1+*; *ura3-1*:: *pRS306 pRS306GAL1pr*-*SNCA*(S129E)-*GFP::URA3+*	[96]
aSyn S129G	W303 *trp1-1:: pRS304 GAL1pr*-*SNCA*(S129G)-*GFP TRP1+*; *ura3-1*:: *pRS306 pRS306 GAL1pr*-*SNCA*(S129G)-*GFP::URA3+*	This study
VSY71*Δpdr5*	VSY71 *PDR5*::KanMX4	This study
VSY72*Δpdr5*	VSY72 *PDR5*::KanMX4	This study
VSY73*Δpdr5*	VSY73 *PDR5*::KanMX4	This study
VSY71*Δatg1*	VSY71 *ATG1*::KanMX4	This study
VSY72*Δatg1*	VSY72 *ATG1*::KanMX4	This study
VSY73*Δatg1*	VSY73 *ATG1*::KanMX4	This study
VSY71*Δatg7*	VSY71 *ATG7*::KanMX4	This study
VSY72*Δatg7*	VSY72 *ATG7*::KanMX4	This study
VSY73*Δatg7*	VSY73 *ATG7*::KanMX4	This study
VSY71 mCherry-Atg8	VSY71 *leu2-3::p305 pATG8* 2XmCherry-*ATG8 LEU2+*	This study
VSY72 mCherry-Atg8	VSY72 *leu2-3::p305 pATG8* 2XmCherry-*ATG8 LEU2+*	This study
VSY73 mCherry-Atg8	VSY73 *leu2-3::p305 pATG8* 2XmCherry-*ATG8 LEU2+*	This study
VSY71 Pup1-RFP	VSY71 *PUP1*:: *p305PUP1*-RFP *LEU2+*	This study
VSY72 Pup1-RFP	VSY72 *PUP1*:: *p305PUP1*-RFP *LEU2+*	This study
VSY73 Pup1-RFP	VSY73 *PUP1*:: *p305PUP1*-RFP *LEU2+*	This study
VSY71 Bre5	VSY71 *pAG303GPD BRE5 HIS3+*	This study
VSY72 Bre5	VSY72 *pAG303GPD BRE5 HIS3+*	This study
VSY73 Bre5	VSY73 *pAG303GPD BRE5 HIS3+*	This study
VSY71 Ypt1	VSY71 *pAG305GPD YPT1 LEU2+*	This study
VSY72 Ypt1	VSY72 *pAG305GPD YPT1 LEU2+*	This study
VSY73 Ypt1	VSY73 *pAG305GPD YPT1 LEU2+*	This study
VSY71 Ykt6	VSY71 *pAG305GPD YKT6 LEU2+*	This study
VSY72 Ykt6	VSY72 *pAG305GPD YKT6 LEU2+*	This study
VSY73 Ykt6	VSY73 *pAG305GPD YKT6 LEU2+*	This study
VSY71 Ubp3	VSY71 *pAG305GPD UBP3 LEU2+*	This study
VSY72 Ubp3	VSY72 *pAG305GPD UBP3 LEU2+*	This study
VSY73 Ubp3	VSY73 *pAG305GPD UBP3 LEU2+*	This study
VSY71 Gyp8	VSY71 *pAG305GPD GYP8 LEU2+*	This study
VSY72 Gyp8	VSY72 *pAG305GPD GYP8 LEU2+*	This study
VSY73 Gyp8	VSY73 *pAG305GPD GYP8 LEU2+*	This study
VSY71 Pmr1	VSY71 *pAG305GPD PMR1 LEU2+*	This study
VSY72 Pmr1	VSY72 *pAG305GPD PMR1 LEU2+*	This study
VSY73 Pmr1	VSY73 *pAG305GPD PMR1 LEU2+*	This study
VSY71 Dcp1-DsRed	VSY71 *pAG305GPD DCP1-*DsRed *LEU2+*	This study
VSY72 Dcp1-DsRed	VSY72 *pAG305GPD DCP1-*DsRed *LEU2+*	This study
VSY73 Dcp1-DsRed	VSY73 *pAG305GPD DCP1-*DsRed *LEU2+*	This study
VSY71 Cerulean-Ypt1	VSY71 *pAG305GPD* Cerulean-*YPT1 LEU2+*	This study
VSY72 Cerulean-Ypt1	VSY72 *pAG305GPD* Cerulean-*YPT1 LEU2+*	This study
VSY73 Cerulean-Ypt1	VSY73 *pAG305GPD* Cerulean-*YPT1 LEU2+*	This study
VSY71 Cerulean-Ypt31	VSY71 *pAG305GPD* Cerulean-*YPT31 LEU2+*	This study
VSY72 Cerulean-Ypt31	VSY72 *pAG305GPD* Cerulean-*YPT31 LEU2+*	This study
VSY73 Cerulean-Ypt31	VSY73 *pAG305GPD* Cerulean-*YPT31 LEU2+*	This study
VSY71 Cerulean-Sec4	VSY71 *pAG305GPD* Cerulean-*SEC4 LEU2+*	This study
VSY72 Cerulean- Sec4	VSY72 *pAG305GPD* Cerulean-*SEC4 LEU2+*	This study
VSY73 Cerulean- Sec4	VSY73 *pAG305GPD* Cerulean-*SEC4 LEU2+*	This study
VSY71 Cerulean-Ypt6	VSY71 *pAG305GPD* Cerulean-*YPT6 LEU2+*	This study
VSY72 Cerulean-Ypt6	VSY72 *pAG305GPD* Cerulean-*YPT6 LEU2+*	This study
VSY73 Cerulean-Ypt6	VSY73 *pAG305GPD* Cerulean-*YPT6 LEU2+*	This study
VSY71 Cerulean-Vps21	VSY71 *pAG305GPD* Cerulean- *VPS21 LEU2+*	This study
VSY72 Cerulean-Vps21	VSY72 *pAG305GPD* Cerulean- *VPS21 LEU2+*	This study
VSY73 Cerulean-Vps21	VSY73 *pAG305GPD* Cerulean- *VPS21 LEU2+*	This study
VSY71 Cerulean-Ypt52	VSY71 *pAG305GPD* Cerulean-*YPT52 LEU2+*	This study
VSY72 Cerulean-Ypt52	VSY72 *pAG305GPD* Cerulean-*YPT52 LEU2+*	This study
VSY73 Cerulean-Ypt52	VSY73 *pAG305GPD* Cerulean-*YPT52 LEU2+*	This study
VSY71 Cerulean-Ypt7	VSY71 *pAG305GPD* Cerulean-*YPT7 LEU2+*	This study
VSY72 Cerulean-Ypt7	VSY72 *pAG305GPD* Cerulean-*YPT7 LEU2+*	This study
VSY73 Cerulean-Ypt7	VSY73 *pAG305GPD* Cerulean-*YPT7 LEU2+*	This study

In contrast, cells expressing the S129A mutant exhibited a longer lag phase of ∼6 hours (Figure S1A). During this period, cells expressing S129A aSyn-GFP lost viability, as indicated by propidium iodide (PI) staining of cells where membrane integrity was compromised (Figure 1B). This was confirmed by spotting assays (Figure S1B). In fact, 6 hours after induction of aSyn expression, 13.6±0.2% of the yeast cells expressing S129A aSyn-GFP were PI positive, compared to only 2.1±0.2% or 2.6±0.5% of cells expressing WT or S129E aSyn-GFP, respectively (Figure 1B). Nonetheless, cells expressing S129A aSyn-GFP were able to adapt and to recover growth ∼7.5 hours after expression induction, with a similar growth rate to those expressing WT or S129E aSyn-GFP (Figure 1A and S1A). These results suggest that yeast cells have mechanisms that allow them to cope with and to recover from toxicity induced by S129A aSyn. Still, cells expressing the mutant S129A aSyn-GFP failed to reach a final OD equivalent to that observed for cells expressing WT or S129E aSyn-GFP (Figure 1A). Altogether, these results demonstrate that expression of S129A aSyn is more toxic for yeast cells than expression of WT or S129E aSyn.

**Figure 2:**
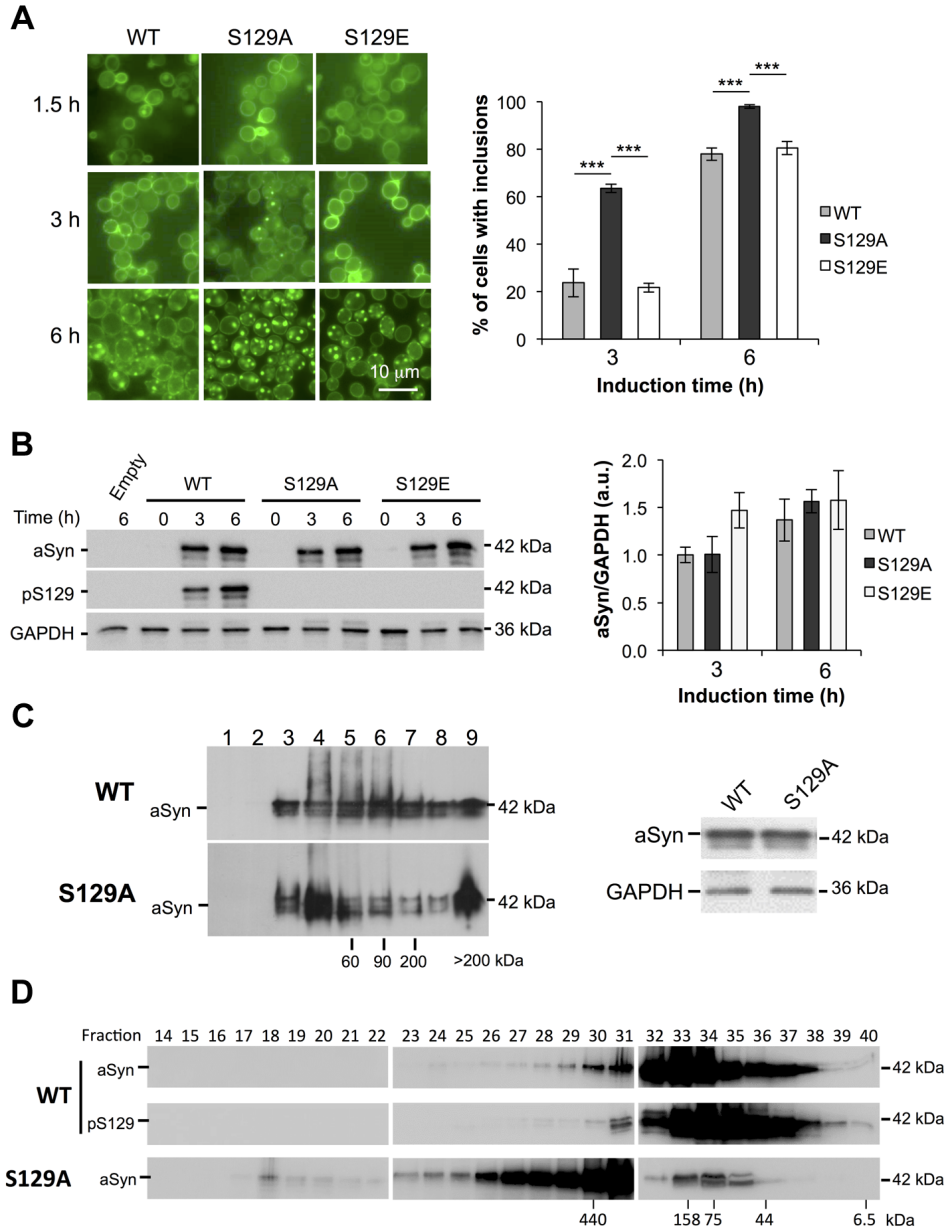
S129A aSyn forms more inclusions and oligomeric species with higher molecular weight than the WT form. (A) Intracellular localization of the WT, S129A or S129E aSyn-GFP (left panel) and percentage of yeast cells containing aSyn-GFP inclusions (right panel), assessed by fluorescence microscopy at the indicated time points after aSyn-GFP expression induction (***p<0.001; one way ANOVA with Bonferroni's multiple comparison test). (B) WT, S129A or S129E aSyn-GFP expression and pS129 levels in yeast cells assessed by western blot analysis of total protein extracts, at the indicated time points after aSyn-GFP expression induction (left panel). Densitometric analysis of the immunodetection of aSyn-GFP relative to the intensity obtained for GAPDH, used as loading control, presented in arbitrary units (a.u.) (right panel). Results shown are from one representative experiment from a total of three independent experiments. (C) The aggregated species formed in yeast cells by the WT and S129A aSyn-GFP were resolved using sucrose gradients after 6 hour of aSyn-GFP expression induction. The resulting fractions were separated on an SDS-page gel followed by immunoblotting with an antibody against aSyn (left panel). The same amount of WT and S129A aSyn-GFP protein was applied to the sucrose gradients, as confirmed by western blot (right panel). (D) The aggregated species formed by WT and S129A aSyn-GFP were also resolved using size exclusion-chromatography (SEC) after 6 hours of aSyn-GFP expression induction. Fractions were collected and separated on SDS-PAGE followed by western blot analyses with an antibody against aSyn or pS129 aSyn. The molecular weights indicated correspond to the calibration curve performed in the SEC, as shown in Figure S3. A representative result is shown from at least three independent experiments. Values represent the mean ± SD.

We then assessed the correlation between cytotoxicity and the subcellular distribution of aSyn-GFP. It was previously described that, initially, aSyn associates with the plasma membrane [16]. Upon increased accumulation of the protein, fluorescent foci appear adjacent to the plasma membrane and, finally, become cytoplasmic inclusions [16], [27], [41]. We imaged cells expressing the WT, S129A or S129E forms of aSyn-GFP by fluorescence microscopy and counted the percentage of cells displaying inclusions at different times after aSyn expression induction (1.5, 3 and 6 hours) (Figure 2A). Interestingly, in addition to being the most toxic, the S129A mutant accelerated inclusion formation. At 3 hours post induction, the percentage of cells displaying inclusions was 3-fold higher for cells expressing the S129A mutant when compared to cells expressing WT protein (63.5±1.8% comparing to 23.7±5.8%) (Figure 2A). After 6 hours of expression induction almost all cells expressing S129A displayed inclusions (98±0.8%). In turn, the S129E mutation did not significantly affect the formation of aSyn-GFP inclusions (Figure 2A).

To confirm the effects observed with the S129A mutant were due to the inability of the protein for being phosphorylated, and not due to structural differences induced by the alanine residue, we also tested an aSyn mutant carrying an S129G substitution. Importantly, the results were identical to those observed with S129A aSyn-GFP (Figure S2). Thus, for subsequent experiments, we continued using the S129A aSyn-GFP phospho-resistant mutant.

We next assessed whether the differences in toxicity and inclusion formation were due to different expression levels of the different variants of aSyn-GFP tested. Using western blot analyses, we found all variants were expressed at similar levels after 6 hours of expression induction (Figure 2B). By using an antibody that specifically recognizes pS129-aSyn, we observed that human aSyn was phosphorylated on S129 residue by endogenous kinases (Figure 2B). Thus, together with the fact that expression of both S129E and WT aSyn-GFP resulted in similar yeast growth phenotypes and percentage of cells with aSyn-GFP inclusions, we concluded that, in our yeast model, the S129E mutation mimics the phosphorylated state of aSyn at S129 (Figure 1 and 2A). For this reason, we continued our study using just WT and S129A aSyn-GFP.

In order to determine the biochemical state of WT and S129A aSyn-GFP we performed centrifugation in sucrose gradients, 6 hours after aSyn expression induction. We found that S129A aSyn-GFP is enriched in fractions corresponding to higher molecular weight species (>200 kDa) (Figure 2C, left panel), and these differences were not due to differences in total protein levels (Figure 2C, right panel).

To further confirm this, we performed size exclusion chromatography (SEC), another widely established method for the biochemical characterization of protein species. For WT aSyn-GFP, we detected species in fractions corresponding to molecular weights of 440 kDa (Figure 2D and S3). Interestingly, for S129A aSyn-GFP, we detected species in fractions corresponding to even higher molecular weights, since these eluted in the void volume of the column (Figure 2D and S3). The distribution of pS129 aSyn-GFP was also analyzed and showed a similar distribution to that of WT aSyn-GFP (Figure 2D). This result convincingly demonstrates, for the first time, that in the yeast model considerable large oligomeric species of aSyn-GFP are formed, an issue that had not been previously resolved in the field.

### Modulation of aSyn S129A toxicity by modifiers of ER-to-Golgi trafficking defects

**Figure 3:**
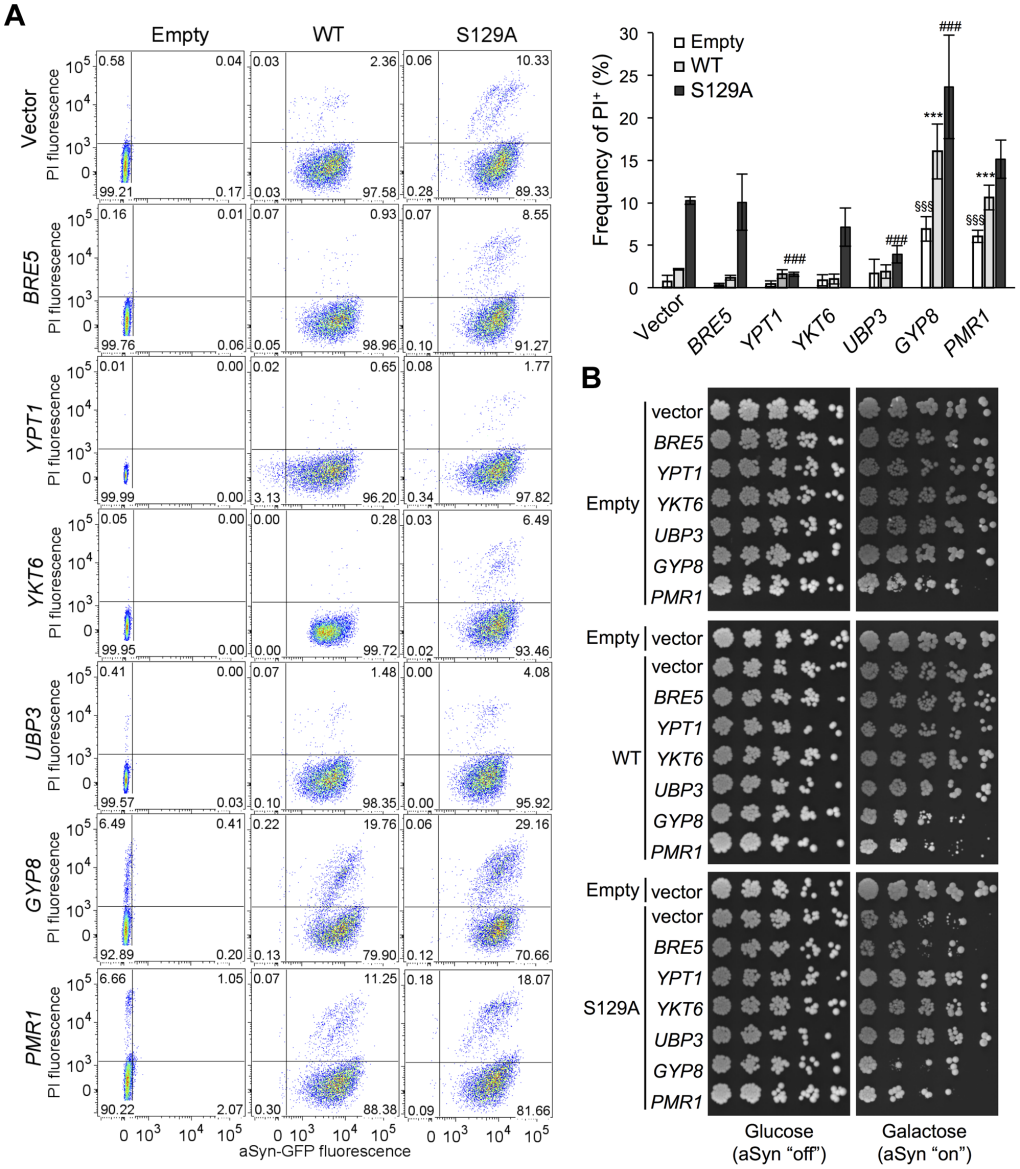
ER-to-Golgi trafficking genes are modifiers of S129A aSyn toxicity. (A) aSyn-GFP versus Propidium Iodide (PI) fluorescence and Frequency of PI positive cells assessed by flow cytometry 6 hours after aSyn expression induction (^§§§^p<0.001 *vs* empty strain; *** p<0.001 *vs* WT; ^###^<0.001 or ^#^p<0.05 *vs* S129A; one way ANOVA with Bonferroni's multiple comparison test). Results shown are from one representative experiment from three independent experiments. Values represent the mean ± SD. (B) Spotting assay of the indicated yeast cells. Cell suspensions were adjusted to the same OD_600nm_, serially diluted and spotted onto the surface of the solid medium containing either glucose (control) or galactose (induced aSyn expression) as carbon source. A representative result is shown from at least three independent experiments.

**Figure 4:**
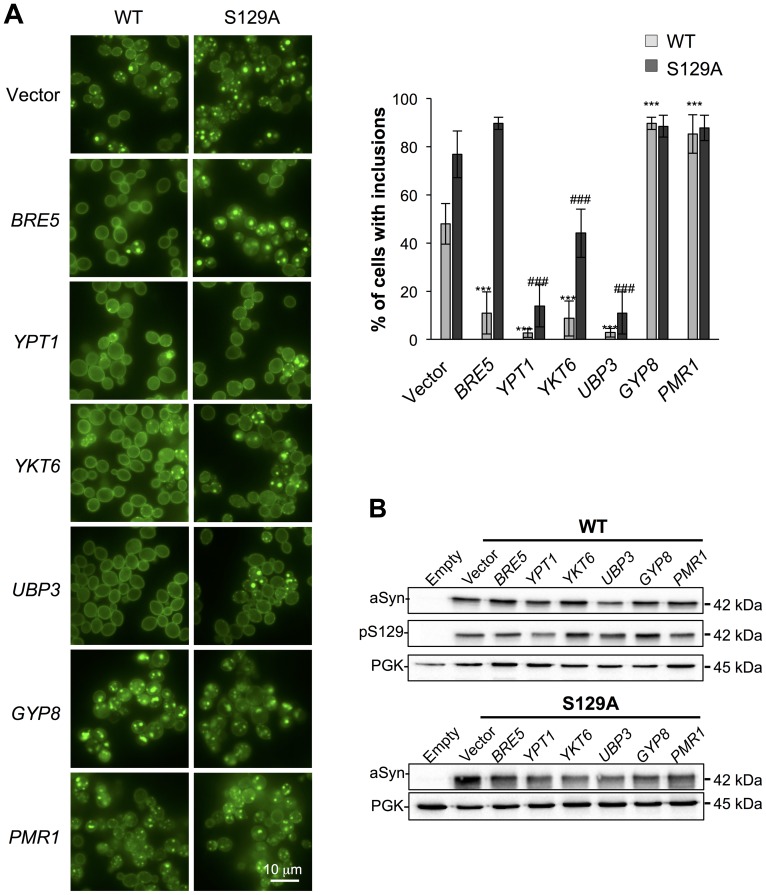
Modulation of ER-to-Golgi trafficking alters S129A aSyn inclusion formation. (A) Intracellular localization of the WT or S129A aSyn-GFP (left panel) and percentage of yeast cells containing aSyn-GFP inclusions (right panel) in the indicated yeast strains, assessed by fluorescence microscopy after 6 hours of aSyn-GFP expression induction (*** p<0.001 *vs* WT; ^###^<0.001 *vs* S129A; one way ANOVA with Bonferroni's multiple comparison test). (B) WT or S129A aSyn-GFP expression and pS129 levels in the indicated yeast strains assessed by western blot analysis of total protein extracts 6 hours after aSyn-GFP expression induction. A representative result is shown from at least three independent experiments. Values represent the mean ± SD.

Several studies demonstrated that aSyn may disrupt multiple intracellular trafficking pathways in yeast [16], [17], [20], [26]-[28], [42], [43]. However, the endoplasmic reticulum (ER)-to-Golgi vesicular trafficking impairment is one of the first defects following induction of aSyn expression [28]. This defect can be rescued by genes promoting ER-to-Golgi trafficking. In addition, genes negatively regulating ER-to-Golgi trafficking enhance aSyn toxicity [28]. To determine if the increased toxicity observed by the blockade of aSyn phosphorylation was also associated with ER-to-Golgi trafficking defects, we tested the effects of previously described suppressors (Ypt1, Ykt6, Ubp3, and Bre5) and enhancers (Gyp8 and Pmr1) of aSyn toxicity in yeast. These modifiers of aSyn toxicity were co-expressed with either WT or S129A aSyn-GFP and the effect on toxicity and inclusion formation was evaluated 6 hours after induction (Figures 3 and 4). As in our yeast model WT aSyn-GFP is moderately toxic, the suppression of toxicity was not remarkable in the spotting assay but became evident when cells were assessed for PI-staining, a more refined method to determine cell viability (Figure 3). Our findings were consistent with what was previously described [28]. Among the suppressors tested, only Bre5 was not able to rescue S129A aSyn-GFP toxicity in both assays (Figure 3). Expression of Ypt1, Ykt6 and Ubp3 significantly reduced the formation of both WT and S129A aSyn-GFP inclusions (Figure 4A) while the overexpression of Bre5 only decreased the formation of WT aSyn-GFP inclusions, and had no effect on S129A inclusion formation (Figure 4A).

Regarding the two enhancers of aSyn toxicity, Gyp8 and Pmr1, expression of an extra copy of these genes exacerbated WT aSyn toxicity as expected [28], and had a similar effect on S129A aSyn (Figure 3). Gyp8 and Pmr1 also increased the formation of aSyn inclusions in WT aSyn-GFP expressing cells without affecting the percentage of cells with S129A aSyn-GFP inclusions, which is already high and therefore precludes the ability to detect an additional increase (Figure 4A).

To determine if the phenotypes observed resulted from an effect of the modifiers on the levels of WT and S129A aSyn-GFP, we preformed western blot analyses. We found that total aSyn and pS129 levels were not affected by the co-expression of the modifiers (Figure 4B).

Altogether, these results indicate that ER-to-Golgi vesicle trafficking defects associated with S129A aSyn toxicity can also be rescued by suppressors of WT aSyn toxicity.

### Blockade of aSyn phosphorylation compromises its degradation

**Figure 5:**
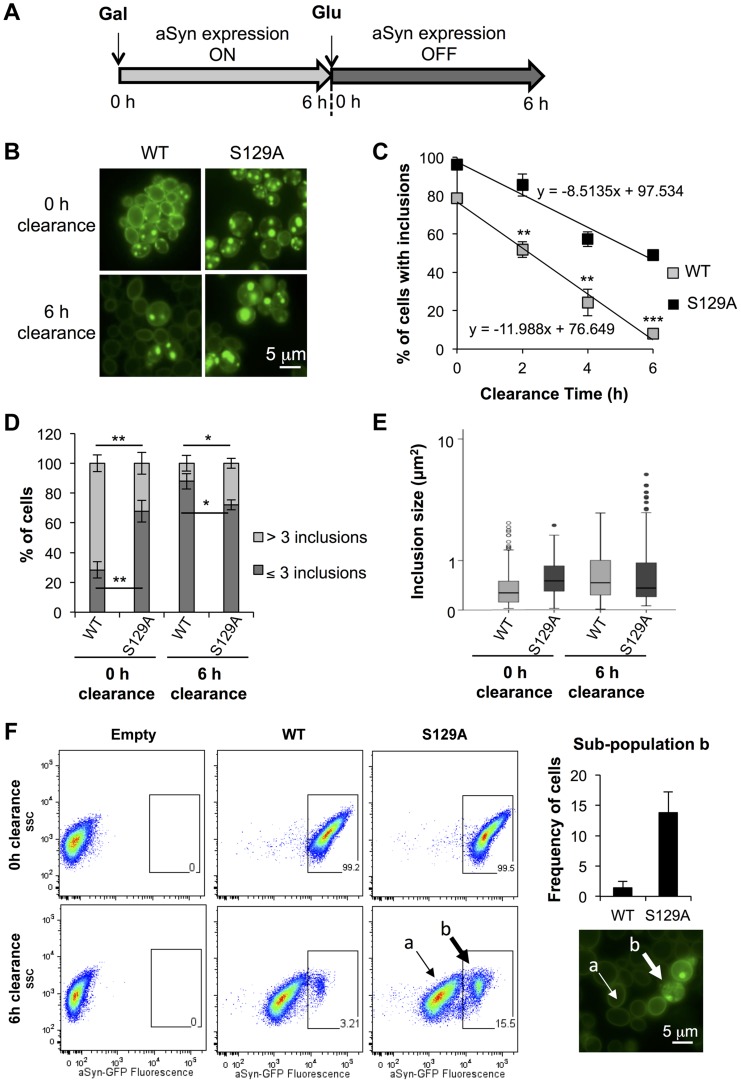
S129A aSyn inclusions clearance is compromised. (A) Schematic representation of the treatments performed: aSyn expression was induced for 6 hours in galactose medium (Gal) and then yeast cells were transferred to glucose medium (Glu) to repress aSyn expression for 6 hours. (B) Intracellular localization of WT or S129A aSyn-GFP and (C) percentage of yeast cells containing aSyn-GFP inclusions, assessed by fluorescence microscopy at the indicated time points of aSyn clearance (***p<0.001, **p<0.01; two-tailed unpaired t-test with Welch's correction). (D) Percentage of cells presenting 3 or less inclusions, or 4 or more inclusions per cell (**p<0.01, *p<0.05; Unpaired two-tailed t-test), and (E) inclusions size (µm^2^) at 0 or 6 hours of aSyn clearance. (F) SSC (side scatter) and aSyn-GFP fluorescence in the indicated yeast cells, assessed by flow cytometry, at 0 and 6 hours after aSyn clearance. A sub-population of yeast cells presenting higher levels of GFP fluorescence is distinguishable after 6 hours of WT or S129A aSyn clearance (left panel). The frequency of cells of this sub-population is represented the in right upper panel. In the right lower panel a fluorescence microscopy picture exemplifies the different GFP intensities observed in aSyn yeast cells after 6 hours of clearance [(a) indicates cells with lower levels of GFP fluorescence and (b) sub-population of yeast cells presenting higher levels of GFP fluorescence]. A representative result is shown from at least three independent experiments. Values represent the mean ± SD.

We postulated that the different biochemical properties might arise from differences in protein clearance of the S129A aSyn-GFP. To test this, aSyn expression was stopped after 6 hours by replacing galactose by glucose (to repress the *GAL1* promoter) and aSyn-GFP clearance was followed for 6 hours (Figure 5A). We found 49±0.4% of cells expressing S129A aSyn-GFP contained inclusions, while only 8±1.2% of the ones expressing the WT form presented aSyn-GFP inclusions, after the clearance period (Figure 5B and C). This corresponds to a reduction of about 50% and 70% when compared to the initial amount of cells with inclusions, respectively (Figure 5C). At 0 hours of clearance (6 hours after aSyn expression induction), S129A expressing cells presented fewer inclusions per cell (Figure 5D). These inclusions were larger and more heterogeneous in size than those formed by WT aSyn-GFP (Figure 5E). After 6 hours of clearance, no significant differences in the size of the inclusions formed by WT or S129A aSyn-GFP were detected (Figure 5E), due to an increase in size of the inclusions formed by WT aSyn-GFP compared to zero of clearance (Figure 5E).

To further investigate how phosphorylation affects aSyn inclusion formation and characteristics, cells were analysed by flow cytometry (Figure 5F). At 0 hours of clearance, both strains presented a homogeneous distribution of the GFP fluorescence showing a single population of cells (Figure 5F). However, after 6 hours of clearance, two populations were visible for both WT and S129A aSyn-GFP, one with weaker (sub-population a) and one with stronger GFP (sub-population b) signal (Figure 5F). We found that the strain expressing S129A aSyn-GFP accumulated more cells in the population displaying stronger GFP signal (11±3.5% versus 1.4±1.0% for WT aSyn-GFP). Together with the results from the fluorescent microscopy, these findings are consistent with cells accumulating inclusions of different sizes, with the larger inclusions displaying stronger GFP signal (Figure 5F).

**Figure 6:**
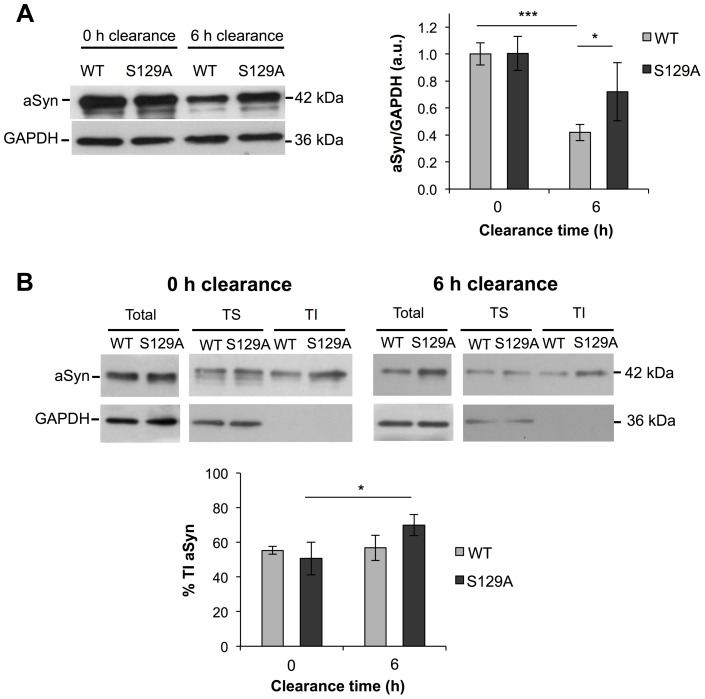
The percentage of Triton-X insoluble S129A aSyn increases during clearance. (A) WT and S129A aSyn-GFP protein levels in yeast cells assessed by western blot analysis of total protein extracts at the indicated time points of aSyn-GFP clearance (left panel). Densitometric analysis of the immunodetection of aSyn relative to the intensity obtained for GAPDH, used as loading control, presented in arbitrary units (a.u.) (right panel) (***p<0.001, *p<0.05; one way ANOVA with Bonferroni's multiple comparison test). (B) Triton-X soluble (TS) and Triton-X insoluble (TI) fractions of aSyn WT or S129A assessed by western blot analysis (upper panel) and determination by densitometric analysis of the percentage of TI aSyn, at the indicated time points of aSyn clearance (lower panel) (*p<0.05; Mann-Whitney test). GAPDH was used as an internal control for the experiment. A representative result is shown from at least four independent experiments. Values represent the mean ± SD.

Next, we evaluated the protein levels of S129A and WT aSyn-GFP at 0 and 6 hours of clearance by western blot analyses. As expected from the results in Figure 2C, no differences were observed at 0 hours of clearance (Figure 6A). In addition, at 0 hours of clearance, both WT and S129A aSyn-expressing cells presented similar levels of Triton-X-Insoluble protein (TI), 55.37±2.23% and 50.69±9.47%, respectively (Figure 6B). However, 6 hours after clearance, the levels of WT aSyn-GFP (41.93±6.04%) were around 32% below those of S129A aSyn-GFP (73.01±19.45%) (Figure 6B), and this was accompanied by a significant increase of the S129A aSyn-GFP in the TI fraction (69.98±6.11%) (Figure 6B). Importantly, during the clearance period, the levels of pS129 aSyn did not change significantly for the WT aSyn-expressing strain (Figure S4).

### S129 phosphorylation modulates aSyn dynamics in inclusions

**Figure 7:**
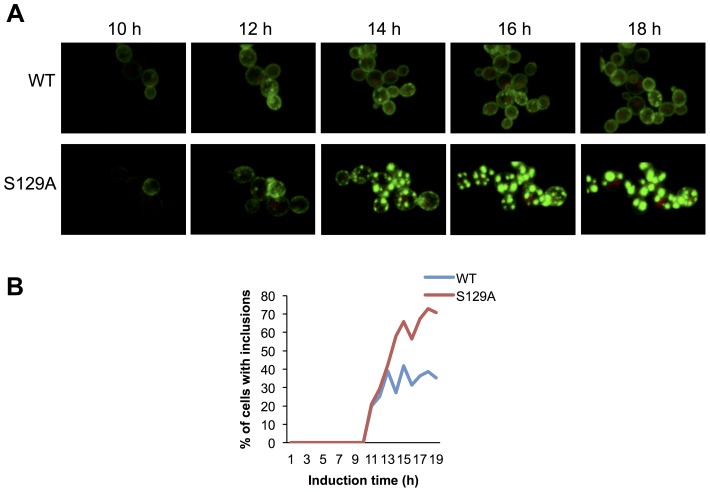
Blocking aSyn phosphorylation reduces membrane interactions and accelerates inclusion formation. (A) Time lapse confocal imaging comparing the distribution of WT or S129A aSyn-GFP at the indicated time points after induction of aSyn expression. Nuclei were visualized by co-expressing NLS-TagRFP657. (B) Percentage of WT and S129A aSyn-GFP expressing cells presenting inclusions over time. n_wt_ = 48 cells, n_S129A_ = 55 cells. Inclusions were recognized by measuring GFP intensity.

Based on the morphological and biochemical differences observed for inclusions formed by WT or S129A aSyn-GFP, we hypothesized that the dynamics of aSyn in the inclusions might differ. To test this, we first used high-resolution 4D imaging in WT and S129A aSyn-expressing cells. Images were acquired every 10 min for 18 hours after induction of aSyn expression (Figure 7 and Supplementary Movie S1 and S2). Initially, WT aSyn-GFP distributed preferentially along the plasma membrane. In contrast, S129A aSyn-GFP formed inclusions sooner than the WT aSyn-GFP (Figure 7). The percentage of cells with inclusions in the population of cells expressing WT or S129A aSyn-GFP was determined and the results confirm that S129A mutation in aSyn promotes inclusions formation in yeast (Figure 7B).

**Figure 8:**
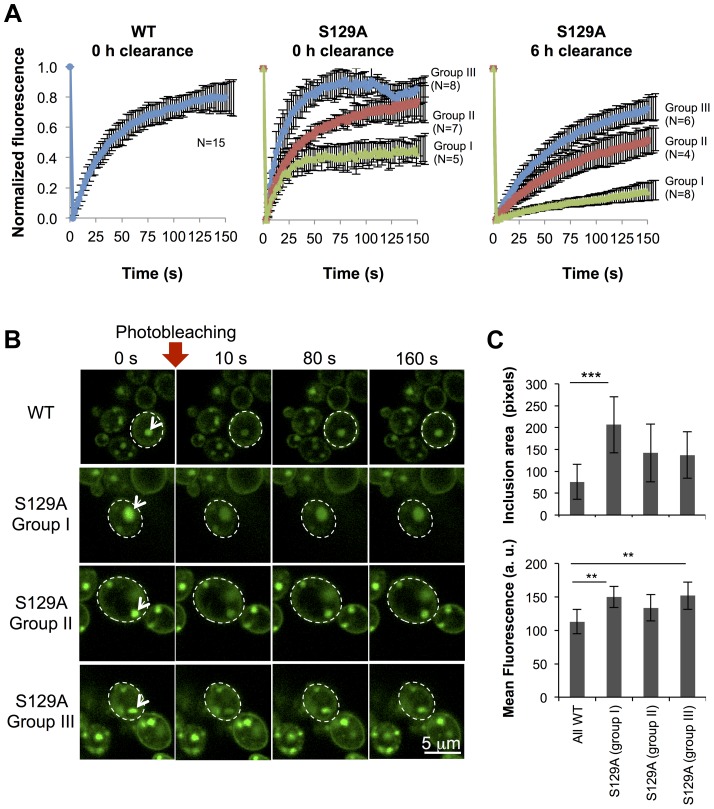
Inclusions formed by S129A aSyn-GFP are more heterogeneous than the ones formed by WT aSyn-GFP. (A) FRAP recovery curves for WT and S129A aSyn-GFP inclusions on yeast cells expressing aSyn forms at the indicated time points. For each strain, at least 15 inclusions in different cells were analyzed. Each plot represents mean ± SD for each time point for all FRAP experiments (for the WT aSyn-GFP expressing cells) or groups of FRAP experiments organized by recovery profiles (for the S129A aSyn-GFP expressing cells). (B) FRAP time lapse recording of a representative inclusion of each group (C) Area (pixels) and fluorescence intensity mean (in arbitrary units, a.u.) of the inclusions analyzed (***p<0.001 and ** p<0.01; one way ANOVA with Bonferroni's multiple comparison test). A representative result is shown from at least three independent experiments. Values represent the mean ± SD.

To further compare the protein dynamics in the inclusions formed by WT or S129A aSyn-GFP, we next used Fluorescence Recovery After Photobleaching (FRAP) and calculated kinetic parameters based on exponential fitting to the FRAP plots (Figure 8). Under the assumption that aSyn-GFP diffusion is very fast compared both to binding and to the timescale of the FRAP experiment, i.e. that binding dominates and diffusion is not detected in the FRAP recovery, an exponential fit to each FRAP recovery curve enables the determination of the aSyn-GFP immobile fraction (IF) and mean residence time (T) (Figure S5A). After 6 hours of aSyn expression induction, the inclusions formed by the WT aSyn-GFP were homogeneous with respect to the relative fluorescence recovery profile (Figure 8A). The residence time for these inclusions was 39.3±0.7 seconds and the immobile fraction, which corresponds to static or long-term bound aSyn-GFP, was 19.3±7.9% (Figure S5A). In contrast, the inclusions formed in cells expressing S129A aSyn-GFP were heterogeneous, and could be distinguished in three main groups, based on their FRAP recovery profiles. In group I the immobile fraction was 55.7±7.5% and the mean residence time was 17.2±0.8 seconds. In group II, the inclusions behaved as the ones formed in cells expressing WT aSyn-GFP, with a mean residence time of 35.2±11.9 seconds and an immobile fraction of 24.1±12.4%. Finally, in group III, inclusions presented a lower mean residence time of 14.9±3.1 seconds and an immobile fraction of 12.7±7.0%. We next determined the mean area and fluorescence intensity of the inclusions analyzed in the FRAP experiments before photobleaching. Inclusions formed by S129A aSyn-GFP were in general larger and presented stronger fluorescence than those formed by WT aSyn-GFP, suggesting the existence of heterogeneity in the inclusions (Figure 8B and C). To test whether inclusion heterogeneity was associated with reduced viability of cells expressing non-phosphorylatable S129A aSyn, we performed FRAP only in PI positive cells containing inclusions. In those cells, the initial fluorescence of the inclusions was not recovered at all and the fluorescence signal was unstable (Figure S6), in contrast to the patterns observed for the WT and S129A inclusions presented in Figure 8. Next, we compared protein dynamics of S129A aSyn-GFP inclusions 6 hours after blocking aSyn expression with that of inclusions at 0 hours of clearance. Again, we distinguished three types of inclusions based on the protein dynamics profiles (Figure 8A). However, after 6 hours of clearance, the immobile fraction as well as the mean residence time increased in the S129A aSyn-GFP inclusions when compared to 0 hours (Figure S5B).

The presence of inclusions with different aSyn-GFP dynamics in the cell led us to hypothesize that the protein was partitioning between distinct subcellular protein quality control compartments. In particular, we hypothesized these compartments might be either the “juxtanuclear quality control” compartment (JUNQ), that is in close proximity to the nucleus and colocalizes with the proteasome, the “insoluble protein deposit” (IPOD), that colocalizes with the autophagic marker Atg8 [44], or P-bodies, cytoplasmic RNA-protein (RNP) granules that contain non-translating mRNAs as a cellular response to stress [45]. To verify these hypotheses, we performed fluorescence microscopy using established sub-cellular markers, namely Atg8 (IPOD), Pup1 (the beta 2 subunit of the 20S proteasome, JUNQ) and Dcp1 (P-Bodies). However, we observed no colocalization between WT or S129A aSyn-GFP with any of these markers (Figure S7).

aSyn inclusions in yeast cells colocalize with diverse trafficking markers including Ypt1 (ER-to-Golgi), Ypt31 (late Golgi), Sec4 (secretory vesicles-to-PM), Ypt6 (endosome-to-Golgi), Vps21 and Ypt52 (early-to-late endosome) and Ypt7 (LE-to-vacuole) [27]. Considering that we observed that S129A aSyn also relates to defects in ER-to-Golgi trafficking, we asked whether blocking pS129 could alter the normal distribution of aSyn inclusions. Thus, we compared the colocalization of WT and S129A aSyn-GFP inclusions using the trafficking markers indicated above. We found that blocking pS129 did not significantly alter the localization of aSyn inclusions (Figure S8).

**Figure 9:**
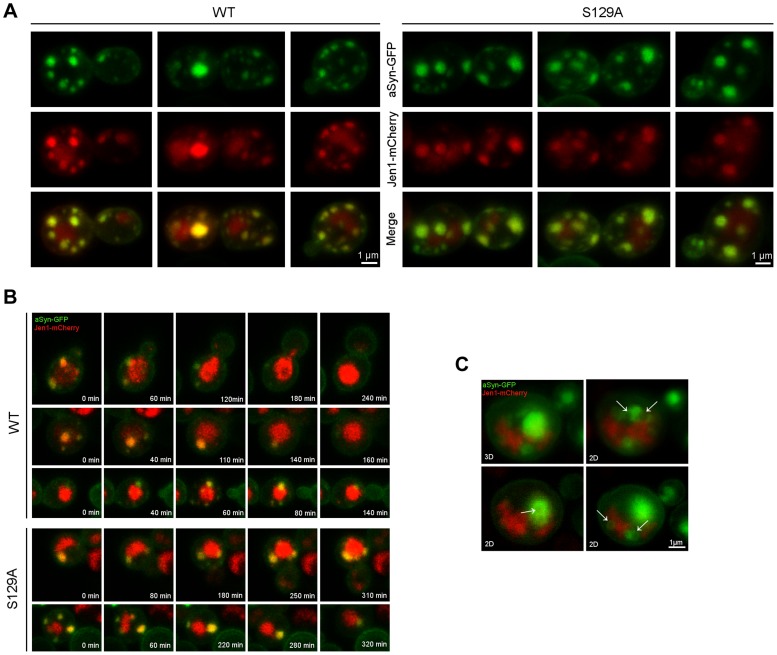
Plasma membrane to vacuole endocytic trafficking of WT and S129A aSyn. (A) aSyn inclusions localize to membrane and endosomal compartments. Cells coexpressing Jen1-mCherry and WT or S129A aSyn-GFP were grown at 30°C in glucose and shifted to galactose for 6 hours. Jen1-mCherry is constitutively synthesized, trafficked to the membrane, and re-absorbed through the endocytic pathway, eventually ending up in the vacuole. Since mCherry is pH stable it is visible in the vacuole. aSyn-GFP inclusions colocalize with Jen1-mCherry in endosome compartments throughout the entire endocytic pathway. (B) aSyn foci cluster with vesicles. Images shown are still frames from a time-lapse series showing internalization of aSyn through the endocytic pathway. In some cases of low level of aSyn aggregation enters the vacuole. As the aggregation level increases, however, aSyn-cotaining vesicles are trapped, likely in late endosomes. (C) aSyn inclusions are present as vesicles or vesicle clusters (arrows). Expression of S129A aSyn-GFP was induced for 8 hours. 3D reconstruction and single z sections (2D) are shown. Each z series was acquired with 0.4 micron step size and 21 total steps.

aSyn has previously been shown to interact with membranes and, at low levels of WT aSyn expression, it localizes mostly to the plasma membrane [16]. Since aSyn also begins to aggregate at the membrane in small vesicles, we sought to investigate its association with the endocytic machinery. In order to visualize plasma membrane to vacuole endocytic trafficking, we used the monocarboxylate-proton symporter Jen1, which undergoes internalization through the endocytic pathway with subsequent vacuolar degradation [46], [47]. At the initial stages of aSyn inclusion formation we observed almost exclusive colocalization of WT and S129A aSyn-GFP inclusions with Jen1, from the plasma membrane, in early endocytic vesicles, to the vacuole (Figure 9 and Supplementary Movie S3 and S4). As aSyn inclusion formation becomes more severe, the vesicles no longer reach the vacuole and accumulate in larger Jen1-positive inclusions, which are likely to be late endosomes (Figure 9). WT aSyn inclusions are present either as single vesicle or smaller clusters, while S129A aSyn inclusions at late stages of aggregation represent large clusters of vesicles (Figure 9).

### Unphosphorylatable aSyn impairs induction of autophagy

Next, we evaluated whether phosphorylation altered the clearance of aSyn in the cell. First, we tested protein degradation by the ubiquitin-proteasome system (UPS). For this, we deleted the *PDR5* gene in the strains expressing aSyn-GFP to ensure chemical inhibition of the proteasome by MG132 [48]. The expression levels of aSyn-GFP in the *Δpdr5* mutants and in the original strains were similar (Figure S9A). Western blot analysis revealed a marked increase in the levels of ubiquitinated proteins in MG132 treated cells, confirming pharmacological proteasome inhibition was achieved (Figure S9B). However, we found that proteasome inhibition did not alter the levels of either WT or S129A aSyn-GFP, nor the percentage of cells displaying aSyn inclusions (Figure S9C). We observed a striking increment in ubiquitinated proteins relative to 3 hours of aSyn clearance (Figure S9B), suggesting the aSyn-induced proteasome inhibition was at least partially reversible.

**Figure 10:**
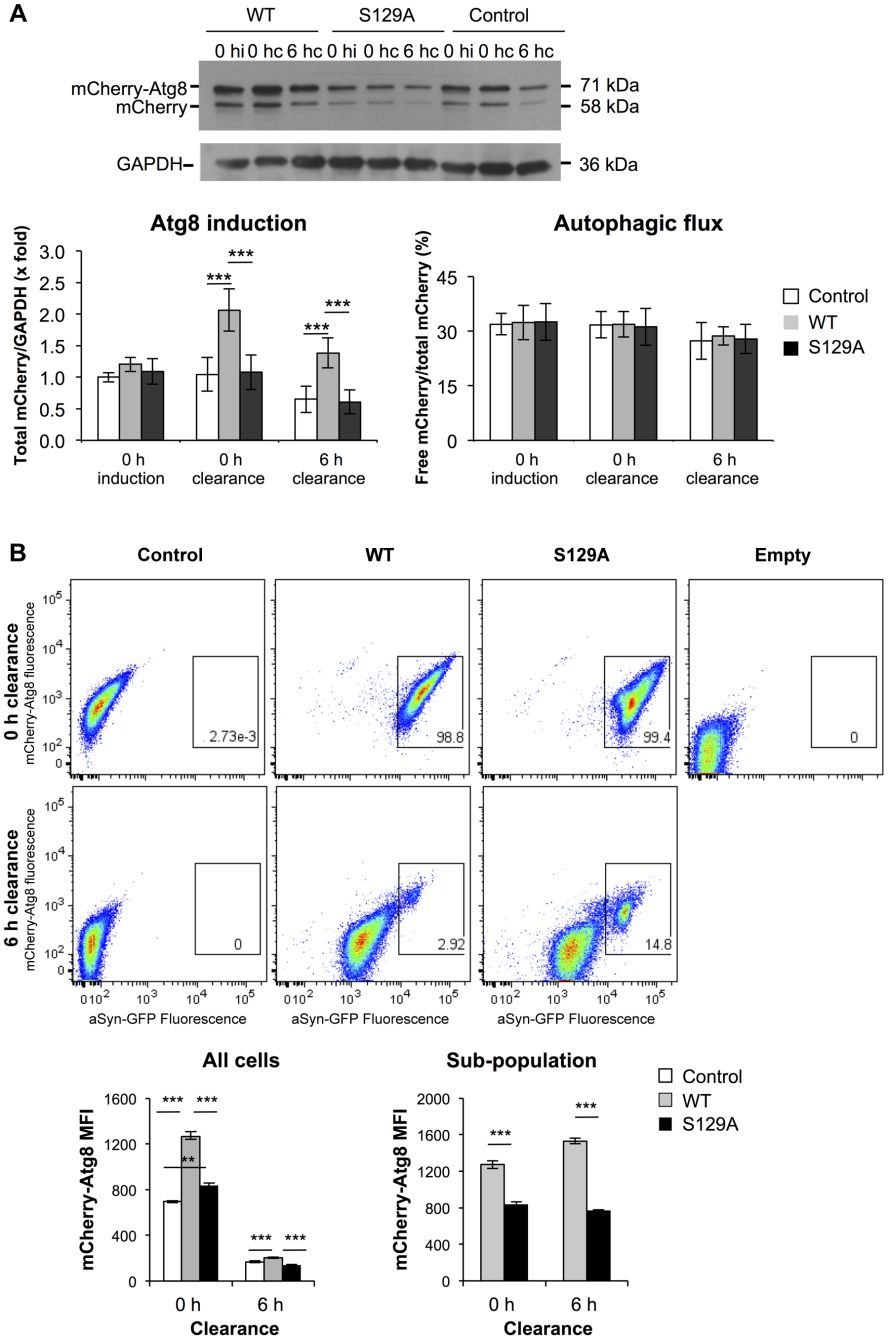
S129A aSyn impairs *ATG8* induction. (A) Autophagy evaluated by mCherry-Atg8 processing assay in yeast cells expressing mCherry-Atg8 under the regulation of the endogenous promoter alone (control) or together with either WT or S129A aSyn, at the indicated time points, assessed by western blot (upper panel) (hi:hours of induction; hc: hours of clearance). *ATG8* induction quantified by the total mCherry signal (mCherry-Atg8 and free mCherry signal, detected with anti-mCherry) (lower left panel); autophagic flux quantified by measuring the vacuolar degradation of the Atg8 domain reporter (ratio of free mCherry to total mCherry signal) (lower right panel). (***p-value<0.001; one way ANOVA with Bonferroni's multiple comparison test). Results shown are from one representative experiment from a total of five independent experiments. (B) aSyn-GFP and mCherry-Atg8 fluorescence intensity in yeast cells expressing mCherry-Atg8 alone (control) or together with either WT or S129A aSyn, at the indicated time points, assessed by flow cytometry (upper panel). mCherry-Atg8 MFI from all cells (lower left panel) and from the indicated sub-population (lower right panel) (***p<0.001, one way ANOVA, with Bonferroni's multiple comparison test). Yeast cells not expressing aSyn-GFP or mCherry-Atg8 were also used as an additional control (empty). A representative result is shown from at least four independent experiments. Values represent the mean ± SD.

Afterwards, we analyzed the contribution of autophagy to the clearance of WT and S129A aSyn-GFP by comparing *ATG8* induction and autophagic flux. We used the mCherry-Atg8 processing assay [49], [50], and inserted the reporter under the control of the endogenous *ATG8* promoter in the genome of the strains expressing WT or S129A aSyn-GFP (Table 1). *ATG8* induction was measured by quantifying the increase of total mCherry signal (mCherry-Atg8 and free mCherry signal) normalized to the loading control (GAPDH) (Figure 10A), reflecting autophagy induction [49], [50]. On the other hand, autophagic flux was quantified by measuring the vacuolar degradation of the Atg8 domain of the reporter (ratio of free mCherry to total mCherry signal) by western blot analysis [49], [50], reflecting the vacuolar transfer and degradation of autophagosomes (Figure 10A). Interestingly, WT aSyn-GFP induced a 2-fold increase in the levels of Atg8 which decreased gradually during the clearance period (Figure 10A). In contrast, the levels of Atg8 remained unaltered throughout induction and clearance in cells expressing S129A aSyn-GFP (Figure 10A). No alterations in autophagic flux were observed with either WT or S129A aSyn-GFP.

Using flow cytometry, we confirmed these results and established a correlation between autophagy induction (measured by mCherry fluorescence intensity) and aSyn-GFP signal (Figure 10B). At 0 hours of clearance the mCherry-Atg8 median fluorescence intensity (MFI) in cells expressing S129A aSyn-GFP were considerable higher than those in cells expressing WT aSyn, and decreased gradually during the clearance period to near basal levels (Figure 10B). These results are consistent with the western blot analysis described above (Figure 10A). As expected, it was visible that, during clearance, the fluorescence of either WT or S129A aSyn-GFP decreases (Figure 10B). However, in both cases a sub-population of cells with larger and brighter inclusions maintained stronger GFP fluorescence after 6 hours of clearance. In this sub-population, higher levels of mCherry-Atg8 were observed both in the cells expressing WT or S129A aSyn-GFP, indicating that autophagy induction is more pronounced in cells with bigger and brighter aSyn inclusions (Figure 10B). However, in this sub-population, the cells expressing S129A aSyn-GFP displayed lower levels of mCherry-Atg8 than cells expressing WT aSyn-GFP both at 0 and 6 hours of clearance (Figure 10B).

**Figure 11:**
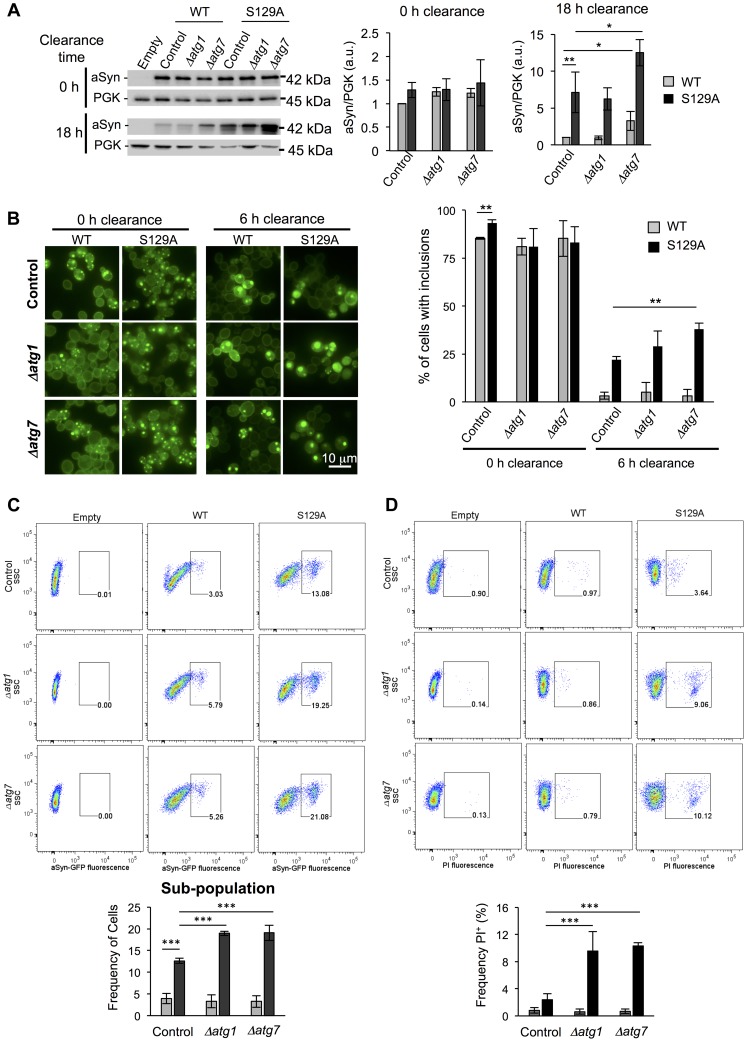
Impairment of autophagy decreases S129A aSyn-GFP clearance and increases toxicity. (A) WT and S129A aSyn-GFP expression levels assessed by western blot analysis of total protein extracts at the indicated time points of aSyn-GFP clearance (left panel). Densitometric analysis of the immunodetection of aSyn relative to the intensity obtained for PGK used as loading control, presented in arbitrary units (a.u.) (right panel). (B) Intracellular localization of WT or S129A aSyn-GFP (left panel) and percentage of cells containing aSyn-GFP inclusions assessed by fluorescence microscopy at the indicated time points of aSyn clearance (right panel). (C) Side scatter (SSC) and aSyn-GFP fluorescence assessed by flow cytometry, at 6 hours of aSyn clearance (upper panel). The frequency of parent of the sub-population of yeast cells presenting higher levels of GFP fluorescence is represented in the lower panel. (D) SSC versus Propidium Iodide (PI) fluorescence and Frequency of PI positive cells assessed by flow cytometry after 6 hours of aSyn clearance. (***p<0.001; **p<0.01, *p<0.05; one way ANOVA with Bonferroni's multiple comparison test). A representative result is shown from at least four independent experiments. Values represent the mean ± SD.

A second line of evidence for the effect of pS129 on aSyn clearance by autophagy was obtained by genetically modulating this pathway. For these studies, we deleted *ATG1* and *ATG7* genes in the WT and S129A aSyn-expressing strains. Atg1 is a kinase playing an important role in autophagy initiation [51] and its mutant is defective in autophagy [52] while Atg7 is an activator of Atg8 and is required for the formation of autophagic bodies [53]. Deletion of *ATG1* and *ATG7* did not significantly affect WT or S129A aSyn-GFP expression levels after 6 hours of induction (Figure 11A). However, when aSyn expression was turned off and clearance was followed during 18 hours, *Δatg7* resulted in higher levels of WT and S129A aSyn-GFP (Figure 11A). Deletion of *ATG1* and *ATG7* significantly increased WT aSyn pS129 levels after 0 hours of clearance (6 hours after aSyn expression induction), an effect that was not observed after 6 hours of clearance (Figure S10).

Deletion of *ATG1* and *ATG7* also significantly increased the percentage of cells displaying aSyn inclusions after 6 hours of clearance (Figure 11B). Flow cytometry experiments confirmed the fluorescence microscopy results, showing a significant increase in the population of cells displaying stronger GFP signal when autophagy was impaired due to *ATG1* or *ATG7* deletion (Figure 11C). Moreover, *Δatg1* and *Δatg7* S129A aSyn-GFP expressing cells also exhibited a higher percentage of PI positive cells, indicating that impairment of autophagy increased S129A aSyn-GFP toxicity, an effect that was not observed for WT aSyn-GFP expressing cells (Figure 11D).

Altogether these results indicate that blocking aSyn phosphorylation impacts on autophagy induction, suggesting cells process phosphorylated aSyn in a distinct way.

## Discussion

Here, we found that WT aSyn-GFP is strongly phosphorylated at S129 by endogenous yeast kinases and S129E mutant aSyn-GFP mimics the behavior of WT protein, in particular with respect to cytotoxicity and inclusion formation. This contrasts with other reports that showed the S129E aSyn-GFP mutant fails to mimic the effect of aSyn S129 phosphorylation [54], [55]. It is possible that the discrepancy is due to differences in the systems used but, importantly, it suggests S129E aSyn mutant might constitute a valid approach for the assessment of the cellular responses involved in the accumulation of phosphorylated aSyn. The S129A mutation was found to promote aSyn fibrillization [10], [56]. However, this was not consensual in all cell and animal models where S129A was expressed. In SH-SY5Y cells, a neuroblastoma cell line with dopaminergic characteristics, S129A aSyn expression reduces inclusion formation [12], while in a *Drosophila* model, S129A aSyn expression results in the accumulation of increased levels of aSyn oligomers, but not of mature fibrils [7]. In our yeast model, expression of the phosphorylation-deficient S129A aSyn-GFP resulted in an exacerbation of aSyn toxicity concomitantly with a reduction in cellular viability and an increase in aSyn inclusion formation. This phenotype does not appear to be related to specific structural consequences of this mutation on aSyn, but rather to the blockade of S129 phosphorylation, as similar results were obtained when S129G aSyn-GFP, a different aSyn phospho-resistant mutant, was expressed.

Despite the accumulating evidence favoring the hypothesis that soluble aSyn oligomers, rather than insoluble protein aggregates, are the cytotoxic species in PD, the question is still unresolved [57], [58]. In yeast, aSyn inclusions were described as clusters of vesicles [26], [27], [42], raising questions about whether aSyn accumulations actually displayed biochemical properties compatible with the formation of protein aggregates. However, at least some of these accumulations are indeed amyloid-like and β-sheeted aggregates, as they react with thioflavin S [41] or thioflavin T [59]. Here, we biochemically characterized the aSyn-GFP species that are formed in yeast cells using two complementary approaches (sucrose gradients and size exclusion chromatography) and clearly demonstrate the formation of large oligomeric species. Moreover, we established a correlation between increased inclusions formation and exacerbation of cytotoxicity and the formation of oligomeric species with higher molecular weight for the S129A aSyn-GFP mutant. Interestingly, we did not observe differences in the TX-insoluble fractions of cells expressing wither WT or S129A mutant aSyn. These results are consistent with other reports where large aSyn soluble oligomers are also considered to constitute the toxic species [60].

Among the various cellular defects that have been implicated in the etiology of synucleinopathies, vesicular trafficking impairment has emerged as a major component of aSyn-dependent toxicity in yeast and in other model organisms [16], [17], [20], [26]–[28], [42], [43]. Both genetic and chemical modulation of vesicular trafficking modulate aSyn toxicity [17], [20], [28]. In this study we show that pS129 blockade exacerbates vesicular trafficking defects that can be relieved by overexpression of *YPT1*, *YKT6* and *UBP3*, genes that increase forward transport between ER and Golgi. Ypt1, the Rab guanosine triphosphatase whose mammalian ortholog Rab1 is able to prevent dopaminergic neuron loss [28], plays an essential role in the tethering and docking of the transport vesicle with the Golgi [61]. Ykt6, the soluble NSF (N-ethylmaleimide–sensitive factor) attachment protein receptor protein (SNARE), increases forward transport by increasing the likelihood of membrane vesicles from the ER tethering to Golgi target membranes [62]. In turn, the ubiquitin protease Ubp3, together with its cofactor Bre5, function to deubiquitinate the COPII coat protein Sec23p, and likewise promote vesicle exit from the ER [63]. Interestingly, the Bre5 cofactor, which also suppresses aSyn toxicity [28], was not able to restore S129A aSyn-GFP induced trafficking defect. This suggests that aSyn phosphorylation may modulate the way the protein interacts with components of the trafficking pathway, as one might expect.

Moreover, *GYP8* and *PMR1* whose overproduction negatively regulates ER-Golgi trafficking, exacerbate S129A aSyn-GFP toxicity. Gyp8, is a negative regulator of Ypt1, that therefore inhibits ER-to-Golgi trafficking [64], while Pmr1 is the major Golgi membrane P-type ATPase ion pump responsible for transporting Ca^2+^ and Mn^2+^ ions into the Golgi apparatus, both of which are important for proper processing and trafficking of proteins through the secretory pathway [65].

Proteostasis is a central concept in the context of several disorders [66]. An imbalance between the rates of protein synthesis, clearance, and aggregation, caused by proteostasis dysfunction, could favor accumulation and/or formation of protein oligomers and inclusions that contribute to cytotoxicity [67]. We found that blocking aSyn phosphorylation impaired the turnover of aSyn. During clearance of S129A aSyn, we observed a significant increase of the TX-insoluble fraction, concomitantly with the attenuation aSyn-induced cytotoxicity. Our observations are consistent with those in a study in *Drosophila* where reduced aSyn toxicity was correlated with an increase in detergent-insoluble aSyn [68] and suggests this may constitute a defense mechanism.

Using high-resolution 4D imaging we found that WT aSyn has a lag phase during which it preferentially associates with the plasma membrane, whereas the S129A forms inclusions almost immediately. Furthermore, blocking aSyn phosphorylation alters the dynamics of aSyn in inclusions suggesting there are distinct populations of aSyn accumulating with different kinetics in inclusions. Based on this, we defined three groups of inclusions based on the recovery after photobleaching.

The presence of an immobile fraction of aSyn is common to all FRAP recovery curves, albeit at different proportions for each type of inclusions. However, blocking aSyn phosphorylation enables the protein to establish transient interactions with inclusions, which occur significantly faster than with WT aSyn.

When the synthesis of aggregation-prone proteins surpasses the degradation capacity of the cell, different quality-control mechanisms that are conserved from yeast to mammalian cells actively sequester aggregated proteins as a protective cellular response [58]. Misfolded aggregated proteins partition between two cellular compartments: the JUNQ and the IPOD compartment, which may serve a protective function and facilitate aggregate clearance. We postulated that the differences in sizes, number and dynamics of the inclusion formed by WT and S129A aSyn might reflect the distribution of aSyn between these compartments where misfolded proteins display distinct relative exchange rates with the soluble cytosolic pool [44], [69], [70]. Non-phosphorylated S129A aSyn accumulating in a less mobile fraction could indicate the protein was localizing to the IPOD, the preferred destination of protein aggregates. Likewise, the S129A aSyn in group III indicated the exchange of soluble aSyn with the cytosolic pool, as described for the JUNQ compartment. Unexpectedly, we found no colocalization of WT or S129A aSyn with neither IPOD nor JUNQ, suggesting that aSyn is not actively sorted to these cellular compartments in yeast. Moreover, we also did not observe colocalization of WT or S129A aSyn-GFP with P-bodies. Alternatively, we did detect colocalization of S129A aSyn-GFP with several vesicular markers including Ypt31 (late Golgi), Sec4 (secretory vesicles-to-PM), Ypt6 (endosome-to-Golgi), Vps21 and Ypt52 [EE-to-late endosome (LE)] and Ypt7 (LE-to-vacuole).

We observed that initial stages of aSyn inclusion formation occur in conjunction with receptor-mediated endocytosis of the plasma membrane symporter Jen1. The alteration in the course of endocytosis caused by the presence of aSyn in endosomal compartments affects trafficking, causes accumulation of vesicles and eventually leads to cell death. The deficiency in the delivery of late endosomes carrying aSyn to the vacuole is more pronounced in the strain expressing S129A aSyn. This prompted us to evaluate the effect of this mutant on endocytosis. The clustering of the endosomal compartment caused by the presence of aSyn on the membrane, slows down endocytic trafficking and eventually blocks internalization of vesicles. While small inclusions of WT and S129A aSyn are cleared, large clusters of S129A aSyn accumulate proximally to the vacuole and, with new cycles of endocytosis, the size of the clusters becomes unbearable for the cell.

Given that early endosome functions are essential for autophagy and for endocytosis-UPS, deregulation of endocytosis by the presence of aSyn in vesicles disrupts not only vesicular trafficking but also major degradation pathways. Vesicles and membranous structures were also observed to accumulate at the periphery of LBs in PD brains [26], reinforcing the relevance of this mechanism in the context of aSyn pathobiology.

Posttranslation modifications modulate the degradation of aggregate-prone proteins by the UPS and/or autophagy. Ubiquitination generally determines whether a protein is degraded via the UPS or autophagy [71]. Interestingly, phosphorylation of mutant huntingtin (Htt), the protein associated with Huntington's disease, was found to precede and regulate additional posttranslational modifications, including ubiquitination, SUMOylation, and acetylation, enhancing its normal clearance by the proteasome and lysosome [72]. In particular, acetylation of mutant huntingtin promotes its targeting to autophagosomes, facilitating its specific degradation by the autophagy/lysosomal pathway [73].

The impact of aSyn S129 phosphorylation on its clearance only started to be investigated recently. In neuronal cell lines, it was observed that proteasome inhibition results in increased levels of pS129 aSyn as an outcome of either increased activity of the kinase(s) involved or decreased phosphatase activity, together with decreased degradation of pS129 aSyn by the proteasome in an ubiquitin-independent manner [74], [75].

Recently, overexpression of GRK6, one of the kinases capable of phosphorylating aSyn at S129 [76], was found to moderately increase aSyn toxicity in a rat model of familial PD [77]. In contrast, another recent study showed that the overexpression of another kinase, the Polo-like kinase 2 (PLK2) [78], is protective by mediating selective autophagy clearance of pS129 aSyn [79]. This apparent discrepancy could be the reflex of the different efficiencies of these kinases to phosphorylate aSyn at S129. In our yeast model, we completely abolished aSyn phosphorylation by replacing S129 with neutral and phosphorylation-resistant amino acids (alanine or glycine). In fact, our results are consistent with those recently reported [79], since we observed that blocking aSyn phosphorylation compromises its degradation. The clearance of the inclusions formed by S129A aSyn was slower than that of inclusions formed by WT aSyn. Moreover, our findings suggest that autophagy is the main mechanism involved in aSyn clearance in our yeast PD model. Whereas the accumulation of WT aSyn led to a marked induction of autophagy, cells expressing the S129A mutant failed to activate this pathway. Thus, we postulate that S129 phosphorylation might constitute a switch to sense and induce the autophagocytic pathway, and that blocking phosphorylation impairs autophagic induction, albeit without altering autophagic flux. Genetic impairment of yeast autophagy by deletion of *ATG1* and *ATG7* did not significantly affect the levels of WT or S129A aSyn after 6 hours of induction suggesting that, at this time point, clearance by autophagy is not superimposed to protein synthesis or that cells could compensate autophagy impairment through other clearance pathways, as suggested by studies performed in other cellular models [38], [39], [80]. However, *ATG7* deletion increases the defect on the clearance of S129A aSyn-GFP but it also has a significant effect on WT aSyn-GFP clearance. This effect could be due to the accumulation of different aSyn species formed by S129A or WT aSyn, as we clearly demonstrated in this study. While soluble and smaller oligomeric species of aSyn could be more easily cleared by the proteasome and CMA, as reported in other models [32], [33], [81], [82], the larger oligomeric species formed by S129A aSyn-GFP could specifically require autophagy function for clearance. Our observation that aSyn accumulation leads to impairment of proteasome function, as previously observed [83], is also consistent with this hypothesis, and this impairment is at least partially reverted when aSyn expression is blocked.

Pharmacological inhibition of the proteasome did not alter the clearance of inclusions in our yeast model, in agreement with previous studies [25]. However, it is now evident that autophagy and proteasome function are deeply interconnected and that inhibition of either one of these pathways results in the compensatory upregulation of the other [38], [80]. Thus, we postulate that upon aSyn-mediated proteasome impairment, autophagy is upregulated as a compensatory mechanism to deal with the excess of aSyn.

In this study, we provide evidence supporting a novel link between aSyn phosphorylation, aggregation and cellular toxicity using a simple but powerful model organism. The finding that the phosphorylation state of aSyn on S129 can have an impact in the ability for cells to clear aSyn inclusions opens novel avenues for intervention in synucleinopathies through the modulation of aSyn phosphorylation.

## Materials and Methods

### Plasmids and yeast strains

The yeast strains used in this work are described in Table 1.

VSY71 to VSY74 contain double genome insertions of *GAL1pr*-*SNCA*(WT, S129A or S129E)-GFP or of the empty vectors and were previously described [42].

Plasmids p304 *GAL1*pr-*SNCA*(S129G)-GFP and p306 *GAL1*pr-*SNCA*(S129G)-GFP were generated by site directed mutagenesis of the corresponding plasmids carrying the WT *SNCA* gene. These plasmids were linearized with EcoRV for integration into W303-1A and W303-1B strains, respectively. The correct insertion of the integrative vectors was verified by PCR and the haploid strains were used to generate a diploid strain by mating. Diploids were selected in minimal medium by URA and TRP prototrophy. Haploid strains carrying the double insertion of *SNCA* S129G were obtained by sporulation and tetrad dissection followed by analysis of auxotrophy and mating type verification. The phenotypic characterization was performed in several haploid strains obtained from dissected spores from independent tetrads and independent mating.

NLS-TagRFP657 was cloned into pESC (*GAL1*pr, LEU2; Stratagene) [84] and used in fluorescence microscopy to visualize cells nuclei.

VSY71 to VSY73 strains with the deleted *PDR5, ATG1 or ATG7* genes, were constructed by gene replacement with a cassette generated by PCR comprising the kanamycin gene (KanMX4) resistance gene flanked by 250 bp gene specific homologous 5′- and 3′- targeting regions. *PDR5*, *ATG1* or *ATG7* deletion was confirmed by PCR (Table 1).

VSY71 to VSY73 2xmCherry-*ATG8* strains contain single genome insertion of the pRS305 2xmCherry-*ATG8* plasmid and were constructed by integration of the EcoRV-linearized pRS305-derived plasmid (Table 1). The 2xmCherry-*ATG8* fragment was excised from the pRS316 2xmCherry-*ATG8* plasmid [85] (a kind gift from Dr. Kuninori Suzuki, Tokyo Institute of Technology), by SacI/KpnI digestion and inserted in the empty pRS305 plasmid, to generate the pRS305 2xmCherry-*ATG8* plasmid.

VSY71 to VSY73 Pup1p-RFP strains contain an integrated RFP (tdimer2;12) at the 3′ end of the Pup1 endogenous loci and were constructed by integration of the EcoNI-linearized pRS305-derived plasmid. The Pup1p-RFP tag was excised from the original plasmid [86] (kindly provided by Dr. Isabelle Sagot, Institut de Biochimie et Génétique Cellulaires), by SacI/HindIII digestion and inserted in the empty pRS305 plasmid, to generate the pRS305 Pup1p-RFP plasmid.

The genes encoding modifiers of aSyn toxicity *YPT1*, *YKT6*, *BRE5*, *UBP3*, *GYP8*, and *PMR1* were kindly provided by Dr. Aaron Gitler, Stanford University, cloned in Gateway entry clones [28] and used to generate expression clones on the pRS based gateway vectors pAG305GAL (*YPT1*, *YKT6*, *UBP3*, *GYP8*, and *PMR1*) or pAG303GAL (*BRE5*) [87]. These plasmids were integrated in the VSY71, VSY72 and VSY73 genome to generate new strains (Table 1).

The vesicles markers Ypt1, Ypt31, Sec4, Ypt6, Vps21, Ypt52 and Ypt7 used on fluorescence microscopy experiments were constructed by Gitler and co-workers [27] and were obtained from Addgene (pAG416GPD-Cerulean-*YPT1*, 18848; pAG416GPD-Cerulean-*YPT31*, 18849; pAG416GPD-Cerulean-*SEC4*, 18844; pAG416GPD-Cerulean-*YPT6*, 18845; pAG416GPD-Cerulean-*VPS21*, 18842; pAG416GPD-Cerulean-*YPT52*, 18843; pAG416GPD-Cerulean-*YPT7*, 18847). These clones were used to generate entry clones by recombination cloning into a Gateway pDONR221 vector (Invitrogen). These clones were then used to generate new integrative vectors in the pAG305 GPD-Cerulean-ccdB vector which were verified by DNA sequencing. These plasmids were integrated in the VSY71, VSY72 and VSY73 genome to generate new strains (Table 1).

The P-bodies marker encoding the gene *DCP1* cloned in pBG1805-*DCP1* was obtained from the Open Biosystems Yeast ORF Collection and used to generate an entry clone into Gateway pDONR221 vector (Invitrogen). This clone was then used to generate a new integrative vector in the pAG305GPD-ccdB-DsRed vector which was verified by DNA sequencing. This plasmid was integrated in the VSY71, VSY72 and VSY73 genome to generate new strains (Table 1). Construction of pESC *JEN1*-mCherry plasmid was performed by ligating BamHI-*JEN1*-XhoI fragment generated by PCR amplification of chromosome DNA with oligonucleotides containing flanking corresponding restriction enzyme sites into BamHI-pESCmCherry-XhoI plasmid. mCherry was subcloned into pESC (LEU) plasmid following XhoI/NdeI restriction sites.

Yeast transformations were carried out using a standard lithium acetate procedure and all the genome insertions were confirmed by two independent PCRs following standard procedures [88].

### Yeast culture conditions and CFUs determination

For aSyn expression induction experiments, yeast cells were pre-grown in YEP-Raffinose (peptone 2%, yeast extract 1%, raffinose 1%) liquid media at 30°C, with orbital agitation (200 rpm) for 24 hours (doubling time: ∼3 hours). After 24 hours, optical density at 600 nm (OD_600 nm_) was measured and yeast cells were diluted to a standardized OD_600 nm_ = 3×10^−3^ (∼2.5×10^5^ cells/mL) in YEP-Raffinose liquid media and grown at 30°C, with orbital agitation (200 rpm). After 24 hours, OD_600 nm_ was measured. The volume of yeast culture needed to inoculate a new culture with an initial standardized OD_600 nm_ = 0.2 (∼7×10^6^ cells/mL) was centrifuged (3000 rpm, at 30°C for 4 min). Cells were then resuspended in YEP-Galactose (peptone 2%, yeast extract 1%, galactose 1%) liquid media and incubated at 30°C, with orbital agitation (200 rpm), for 6 hours. The cell viability was assessed by counting CFUs after incubation of culture aliquots for 2 days at 30°C on YEP-glucose agar plates.

For spot assays cell suspensions were adjusted to OD_600nm_ = 0.05±0.005 and used to prepare 1/3 serial dilutions that were applied as spots (4 µl) onto the surface of the YPD rich medium used as control or YEP-Galactose medium and incubated at 30°C for 2–3 days.

For aSyn clearance experiments, OD_600 nm_ of the 6 hours induced cultures was measured and the volume of yeast culture needed to inoculate a new culture with an initial standardized OD_600 nm_ = 0.2 (∼7×10^6^ cells/ml) was centrifuged (3000 rpm, at 30°C for 4 min). Cells were washed in PBS and resuspended in YEP-Glucose (peptone 2%, yeast extract 1%, glucose 2%) liquid media and incubated at 30°C, with orbital agitation (200 rpm), for 6 hours.

For fluorescence microscopy or flow cytometry analysis, adenine was added to the growth media at a final concentration of 0.16 mg/mL to avoid background interactions by the red pigment production due to *ade2* auxotrophic marker of the used yeast strain. Adenine supplementation did not alter growth phenotypes of the tested yeast strains.

### Flow cytometry

Yeast cell membrane integrity was evaluated with PI staining using a BD LSR Fortessa. Yeast cells were incubated with PI 5 µg/mL for 15 min. As a positive control, cells boiled for 10 min were used (data not shown).

Autophagy induction was determined measuring fluorescence intensity of mCherry-Atg8 under the regulation of the natural promoter [85] in cells co-expressing WT or S129A aSyn-GFP, using a BD FACSAria III equipped with a 561 nm laser for excitation and a 600 LP mirror in conjunction with a 610/20 BP filter for detection (BD Biosciences, San Jose, CA). Fluorescence intensity of WT or S129A aSyn-GFP was measured in simultaneous using a 488 nm laser for excitation and a 502 LP mirror in conjunction with a 530/30 BP filter for detection (BD Biosciences, San Jose, CA).

A minimum of 10.000 events were collected for each experiment. Data analysis was performed using FlowJo software (Tree Star Inc., Ahsland, OR, USA). Results were expressed as median fluorescence intensity (MFI) of a molecule.

### Protein extraction and western blot analysis

For total protein extraction, yeast cells were lysed in Tris-HCl buffer pH 7.6 supplemented with protease and phosphatases inhibitor cocktail (Roche, Mannheim, Germany), with glass beads (3 cycles of 30 seconds in the beadbeater and 1 min on ice). Cell debris was removed with a smooth centrifugation (700 g, 3 min, 4°C) and the supernatant was collected. The supernatant was sonicated (10 seconds at 10 mA, Soniprep 150 from Sanyo). Protein concentration was determined using the BCA protein assay kit (Thermo Fisher Scientific Inc, Illinois, USA). The same amount of total protein was loaded in the SDS-PAGE for the detection of mCherry-Atg8 levels. As WT and mutant aSyn expression cells exhibit slightly distinct growth rates, equal volumes of total protein, corresponding to the same number of cells (normalized based on OD_600 nm_) were applied to the SDS-PAGE for aSyn quantification in induction, clearance and proteasome pharmacological inhibition experiments, in order to avoid bias in the protein measurement levels due to a cell dilution effect.

Protein sample buffer (200 mM Tris-HCl pH 6.8, 6% 2-mercaptoethanol, 8% SDS, 40% glycerol, 0.4% bromophenol blue) was added to each protein sample and heated for 10 min at 100°C before acrylamide gel loading. Protein samples were run in SDS-PAGE and were transferred to a nitrocellulose membrane using a Trans-Blot Turbo transfer system (Bio-Rad), as specified by the manufacturer. Immunoblotting was performed following standard procedures with the listed antibodies: aSyn (BD Transduction Laboratories, San Jose, CA, USA), pS129-aSyn (Wako Chemicals USA, Inc., Richmond VA, USA) and DsRed (Clontech Laboratories, Inc. USA). GAPDH (Ambion, Cambridgeshire, UK) or PGK (Life Technologies, Grand Island, NY, USA) were used as loading control.

The band intensity of the different immunoblots signals was estimated using ImageJ software (NIH, Bethesda, MD) and normalized against the corresponding GAPDH or PGK signal. In particular, aSyn levels were determined by calculating the ratio between aSyn/GAPDH and normalized to the control (mean ± SD); pS129-aSyn levels were determined by doing the ratio between both values: (pS129/GAPDH)/(aSyn/GAPDH) and normalized to the control (mean ± SD). Atg8 induction was quantified by the determination of the fold increase of total mCherry signal (mCherry-Atg8 and free mCherry signal, detected with anti-DsRed) normalized to GAPDH [89]; autophagic flux was quantified by measuring the vacuolar degradation of the Atg8 domain reporter (ratio of free mCherry to total mCherry signal) [50].

### Triton soluble and insoluble fractions

Total protein was extracted and quantified with the BCA protein assay kit (Thermo Fisher Scientific Inc, Illinois, USA). 200 µg of total protein was incubated with 1% Triton X-100 on ice, for 30 min. Protein fractions were separated by centrifugation at 15000 g for 60 min at 4°C. The top soluble protein fraction (Triton-soluble, TS) was collected and the insoluble protein fraction (Triton-insoluble, TI) pellet was resuspended in 40 µl of 2% SDS Tris-HCl buffer pH 7.4 by pipetting and subsequent sonication (10 seconds). Equal volumes of TS and TI were loaded and resolved by SDS-PAGE.

### Sucrose gradient and size exclusion chromatography

Total protein from cells expressing WT or S129A aSyn was obtained and applied on a 5 to 30% sucrose gradient as described before [90], [91]. Fractions were collected, precipitated for 4 hours at 4°C in trichloroacetic acid, washed in acetone three times and suspended in protein sample buffer (0.5 M Tris-HCl, pH 6.8, Glycerol, 10% SDS, 0.1% Bromophenol Blue). Proteins were resolved by SDS-PAGE and estimation of the molecular sizes for each fraction was previously described [92].

Size exclusion-fast protein liquid chromatography (SEC-FPLC) was performed with total protein lysates from cells expressing WT or S129A aSyn-GFP extracted as described for sucrose gradient [90], [91], centrifuged at 16000 g for 4 min and filter with 0.45 µM PVDF (Whatman) to remove any insoluble particles. Samples (3 mg of protein in final volume of 500 µL) were analyzed on a Superose 6 10/300 GL column (GE Healthcare, Uppsala, Sweden) using a FPLC system with UV-M II detector (GE Healthcare, Uppsala, Sweden). The samples were eluted with 50 mM ammonium acetate, pH 7.4 at a flow rate of 500 µL/min and the UV absorbance was monitored at 280 nm. To estimate the molecular weight of the protein samples, High Molecular Weight and Low Molecular Weight gel filtration calibration kits were used (GE Healthcare, Uppsala, Sweden). Fractions of 500 µL were collected, precipitated overnight at 4°C in trichloroacetic acid, washed in acetone three times and resuspended in protein sample buffer (0.5 M Tris-HCl, pH 6.8, Glycerol, 10% SDS, 0.1% Bromophenol Blue), and were resolved by SDS-PAGE.

### Fluorescence 3D time-lapse (4D imaging)

For time-lapse imaging, VSY yeast cells transformed with pESC-Leu *GAL1*pr NLS-TagRFP657 were pre-grown overnight in Synthetic complete (SC) medium without leucine (SC-Leu) raffinose liquid media at 30°C, with orbital agitation (200 rpm). OD_600 nm_ was measured and yeast cells were diluted to a standardized OD_600 nm_ = 0.8 (∼2.4×10^7^ cells/ml) in SC-Leu raffinose liquid media and grown at 30°C, with orbital agitation (200 rpm). After 6 hours, cells were seeded on concanavalin A-coated 4-well microscope plates (Greiner Bio-One GmbH) for about 10 min. Then all media was replaced by SC-Leu Galactose for aSyn and NLS-TagRFP657 expression induction. Confocal 3D movies were acquired using a dual point-scanning Nikon A1R-si microscope equipped with a PInano Piezo stage (MCL), using a 60x PlanApo IR water objective NA 1.27 and PlanApo VC oil objective 960x) NA 1.40, 0.35 micron slices, and 0.5% laser power (from 5 mW 488 laser and 40 mW 561 laser). Prior to imaging the point-spread function was visualized with 100 nm fluorescence beads in order to adjust the correction ring of the objective to the coverslip thickness. Movies were acquired in resonant-scanning mode. Z-stacks were acquired every 10 min for 18 hours with some exceptions. Each z-series was acquired with 0.35 micron step size and 30 total steps, images - in galvano scanning mode (0.4 micron slices). Image processing was performed using NIS-Elements software.

### Fluorescence microscopy and FRAP experiments

The percentage of cells with aSyn inclusions, number of aSyn inclusions per cell and size of aSyn inclusions were determined by fluorescence microscopy using a Zeiss Axiovert 200 M (Carl Zeiss) widefield fluorescence microscope equipped with a cooled CCD camera (Roper Scientific Coolsnap HQ) to acquire images containing at least 700 cells per strain, which were then manually counted using ImageJ. Colocalization studies were performed with a Zeiss Axiovert 200 M (Carl Zeiss) widefield fluorescence microscope equipped with a cooled CCD camera (Roper Scientific Coolsnap HQ) or a Zeiss LSM 710 inverted laser scanning confocal microscope (Carl Zeiss) using a Plan-Apochromat 63x/1.4 oil immersion objective. EGFP fluorescence was detected using the 488 nm laser line of an Ar laser (25 mW nominal output) and a custom wavelength detection window set to 493–556 nm. mCherry and RFP fluorescence were detected using a 561 nm DPSS laser (15 mW) and a custom detection window set to 569–797 nm.

Yeast cells were grown as described above. At the indicated time points cells were collected by centrifugation and resuspended in PBS and 0.5% low melting agarose on a microscope slide.

FRAP experiments were performed using a Zeiss LSM 710 inverted laser scanning confocal microscope equipped with a large incubator (Pecon, Erbach, Germany) maintained at 30°C. Images were acquired using a PlanApochromat 63x/1.4 objective. A series of 80 z-stacks consisting of 5 different focal planes spaced 0.7 µm apart (frame size 512×512, pixel width 91 nm and pixel time 4.44 µs) were acquired at intervals of 2 seconds with pinhole set to 1 Airy unit. In each FRAP experiment a single inclusion, focused at the central focal plane of the z-stack, was bleached using the 488 nm laser line at 100% laser transmission on a circular region of interest (ROI) with a diameter of 8 pixels (0.35 µm radius) for 32 ms. For imaging, the transmission of the 488 nm laser was set to 0.3% of the bleach intensity.

Image processing and fluorescence intensity measurements were performed in ImageJ using an in-house developed macro to extract the average fluorescence in the bleached area *I(t)* at the central plane of the z-stack at each time point *t* and the average cellular fluorescence intensity *T(t)* which was used to normalize FRAP recovery curves as described previously [93]. 
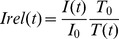
where *T_0_* is the fluorescence in the total cell area before bleaching and *I_0_* is the fluorescence in the bleached region before bleaching. Post-bleach values were additionally set to zero to facilitate comparison of the curves.

FRAP recovery curves were fit to a single-exponential curve assuming a binding-dominant (diffusion-uncoupled) regime where protein diffusion occurs much faster than binding kinetics [94]. 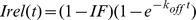
where *IF* is the immobile fraction and *k_off_* is the off rate of binding which can be used to determine the mean residence time *T = 1/k_off_.* Curve fitting was performed on OriginPro 8 (OriginLab Corporation).

### Statistical analysis

Statistical analysis were performed using unpaired tow-tailed t-test; one way ANOVA with Bonferroni's or Tuckey multiple comparison test; two-tailed unpaired t-test with Welch's correction; two-tailed Mann-Whitney test, where appropriate. P-value ≤0.05 was considered statistically significant. Statistics were performed using Prism 5 and SigmaStat (GraphPad Software Inc.).

## Supporting Information

Figure S1 S129A aSyn is more toxic than WT aSyn. (A) Cell viability determined by CFUs during the initial time points of the growth curve (shown in Figure 1A) of yeast cells expressing either WT (□), S129A (▴) or S129E (○) aSyn-GFP, compared to cells that are not expressing the human protein (◊). Cells used as inoculum were exponential-phase cells cultivated in raffinose medium that at time zero were transferred to galactose medium to induce aSyn expression. Values are representative from three independent experiments. (B) Spotting assay of the indicated yeast cells. The cell suspensions with adjusted OD_600nm_ were serially diluted and spotted onto the surface of solid medium containing either glucose (control) or galactose (induced aSyn expression) as carbon source. A representative result is shown from at least three independent experiments.
(TIFF)

Figure S2 S129G aSyn behaves similarly to S129A asyn. (A) Spotting assay of the indicated yeast cells. The cell suspensions with adjusted OD_600nm_ were serially diluted and spotted onto the surface of solid medium containing either glucose (control) or galactose (induced aSyn expression) as carbon source. Results shown are from one representative experiment from at least three independent experiments. (B) WT, S129A or S129G aSyn-GFP expression and pS129 levels in yeast cells assessed by western blot analysis of total protein extracts 6 hours after aSyn-GFP expression induction. PGK was used as loading control. (C) Intracellular localization of the WT, S129A or S129G aSyn-GFP (left panel) and percentage of yeast cells containing aSyn inclusions (right panel), after 6 hours of aSyn expression induction, assessed by fluorescence microscopy (**p<0.01; one way ANOVA and post-hoc Tukey test). Results shown are from one representative experiment from at least three independent experiments. Values represent the mean ± SD.
(TIFF)

Figure S3 Elution profiles of SEC from total protein extracts of cells expressing WT or S129A aSyn-GFP. Equal amounts of total protein (∼3 mg) from cells not expressing aSyn (empty) or expressing either WT or S129A aSyn-GFP were separated in a Superose 6 10/300 GL column. A calibration curve for SEC was performed using protein molecular weight markers (ferritin, 440 kDa; aldolase, 158 kDa; conalbumin, 75 kDa; ovalbumin, 44 kDa, and aprotinin, 6.5 kDa). The elution time of the protein markers is indicated in the figure. Results shown are from one representative experiment from at least three independent experiments. Values represent the mean ± SD.
(TIFF)

Figure S4 S129 phosphorylation levels of WT aSyn are not altered during aSyn clearance. WT aSyn and pS129 levels at the indicated time points of aSyn clearance (left panel). Densitometric analysis of the pS129-aSyn levels by determining the ratio of the pS129 and the total levels of aSyn (pS129/PGK)/(aSyn/PGK) and normalized to the control. Results shown are from one representative experiment from at least four independent experiments. Values represent the mean ± SD.
(TIFF)

Figure S5 aSyn protein dynamics in inclusions. FRAP recovery curves of the aSyn WT and S129A inclusions shown in Figure 5 are well fit with a single exponential curve. FRAP experiments were performed at (A) 0 hours and (B) at 6 hours of clearance. The aSyn immobile fraction (IF) and the aSyn mean residence time (T) values were calculated from the single exponential fit. Values represent the mean ± SD.
(TIFF)

Figure S6 aSyn inclusions from PI-positive cells expressing S129A aSyn do not recover after photobleaching. (A) Time lapse recording of the fluorescence recovery after photobleaching of aSyn inclusions in three representative PI positively marked cells expressing aSyn S129A after 6 hours of expression induction and the corresponding (B) FRAP recovery curve of cells shown in (A).
(TIFF)

Figure S7 Inclusions formed by WT or S129A aSyn do not colocalize with IPOD, JUNQ or P-bodies markers. Confocal microscopy images of cells co-expressing either WT or S129A aSyn-GFP and mCherry-Atg8, Pup8-RFP or Dcp1-DsRed after 6 hours of aSyn expression induction. Results shown are from one representative experiment from at least three independent experiments.
(TIFF)

Figure S8 Inclusions formed by WT or S129A aSyn colocalize with vesicular trafficking markers. Fluorescence microscopy images of cells co-expressing either WT or S129A aSyn-GFP, and the indicated vesicular trafficking markers with N-terminal fusions with cerulean, after 6 hours of aSyn expression induction. Results shown are from one representative experiment from at least three independent experiments.
(TIFF)

Figure S9 The proteasome is not involved in the clearance of S129A aSyn-GFP. (A) WT, S129A aSyn-GFP expression levels in WT or *Δpdr5* yeast cells assessed by western blot analysis of total protein extracts 6 hours after aSyn-GFP expression induction. (B) Percentage of *Δpdr5* cells with WT or S129A aSyn-GFP inclusions, before and after 3 hours of clearance in the presence of MG132 in DMSO or only DMSO. (C) WT and S129A aSyn-GFP expression levels at the same time points and subject to the same treatments as in (B), determined by western blot analysis of protein total extracts from *Δpdr5* yeast cells. The total levels of ubiquitinated proteins were also evaluated by immunoblotting to confirm the effectiveness of the MG132 as proteasome activity inhibitor. Results shown are from one representative experiment from at least three independent experiments. Values represent the mean ± SD.
(TIFF)

Figure S10 Impairment of autophagy do not affect aSyn S129 phosphorylation. WT aSyn-GFP pS129 levels assessed by western blot analysis of total protein extracts at the indicated time points of aSyn-GFP clearance (left panel). Densitometric analysis of the pS129-aSyn levels by determining the ratio between pS129 and the total levels of aSyn (pS129/PGK)/(aSyn/PGK) and normalized to the control (right panel) (*p<0.05; one way ANOVA with Bonferroni's multiple comparison test). A representative result is shown from at least three independent experiments. Values represent the mean ± SD.
(TIFF)

Movie S1 High-resolution 4D imaging of yeast cells expressing WT aSyn-GFP. Nuclei are marked with NLS-TagRFP657. Images were acquired every 10 min for 18 hours after induction of aSyn expression.
(AVI)

Movie S2 High-resolution 4D imaging of yeast cells expressing S129A aSyn-GFP. Nuclei are marked with NLS-TagRFP657. Images were acquired every 10 min for 18 hours after induction of aSyn expression.
(AVI)

Movie S3 High-resolution 4D imaging of yeast cells expressing WT aSyn-GFP and Jen1-mCherry. Images were acquired every 10 min after induction of aSyn expression.
(AVI)

Movie S4 High-resolution 4D imaging of yeast cells expressing WT aSyn-GFP) and Jen1-mCherry). Images were acquired every 10 min after induction of aSyn expression.
(AVI)
