# Supplementary figures and images for: Phosphorylation Modulates Clearance of Alpha-Synuclein Inclusions in a Yeast Model of Parkinson's Disease
Source: PLoS Genet. 2014 May 8;10(5):e1004302. doi: 10.1371/journal.pgen.1004302 (PMC4014446; doi:10.1371/journal.pgen.1004302)

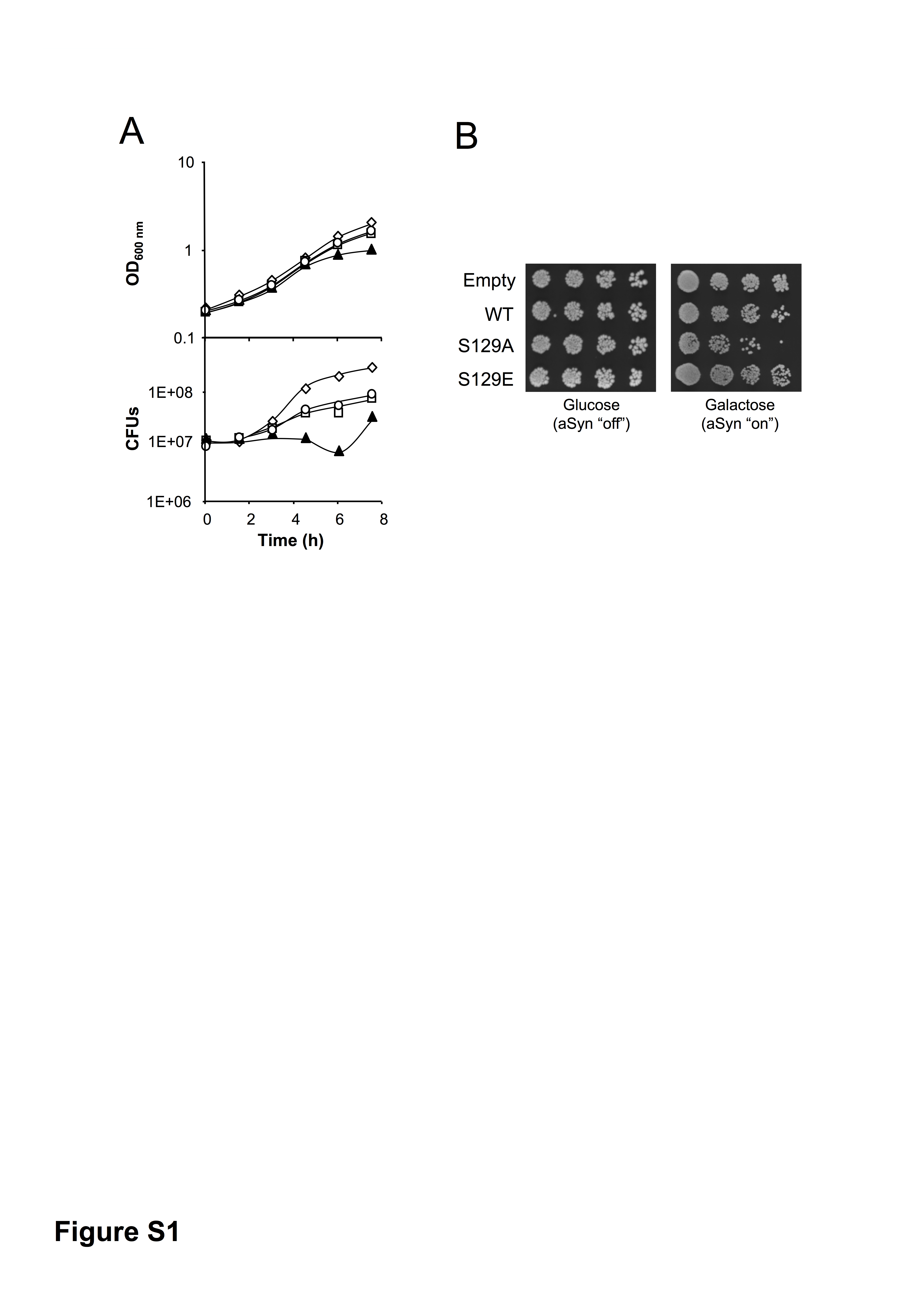

Supplement: Figure S1 — S129A aSyn is more toxic than WT aSyn. (A) Cell viability determined by CFUs during the initial time points of the growth curve (shown in Figure 1A) of yeast cells expressing either WT (□), S129A (▴) or S129E (○) aSyn-GFP, compared to cells that are not expressing the human protein (◊). Cells used as inoculum were exponential-phase cells cultivated in raffinose medium that at time zero were transferred to galactose medium to induce aSyn expression. Values are representative from three independent experiments. (B) Spotting assay of the indicated yeast cells. The cell suspensions with adjusted OD600nm were serially diluted and spotted onto the surface of solid medium containing either glucose (control) or galactose (induced aSyn expression) as carbon source. A representative result is shown from at least three independent experiments. (TIFF) [file pgen.1004302.s001.tiff]

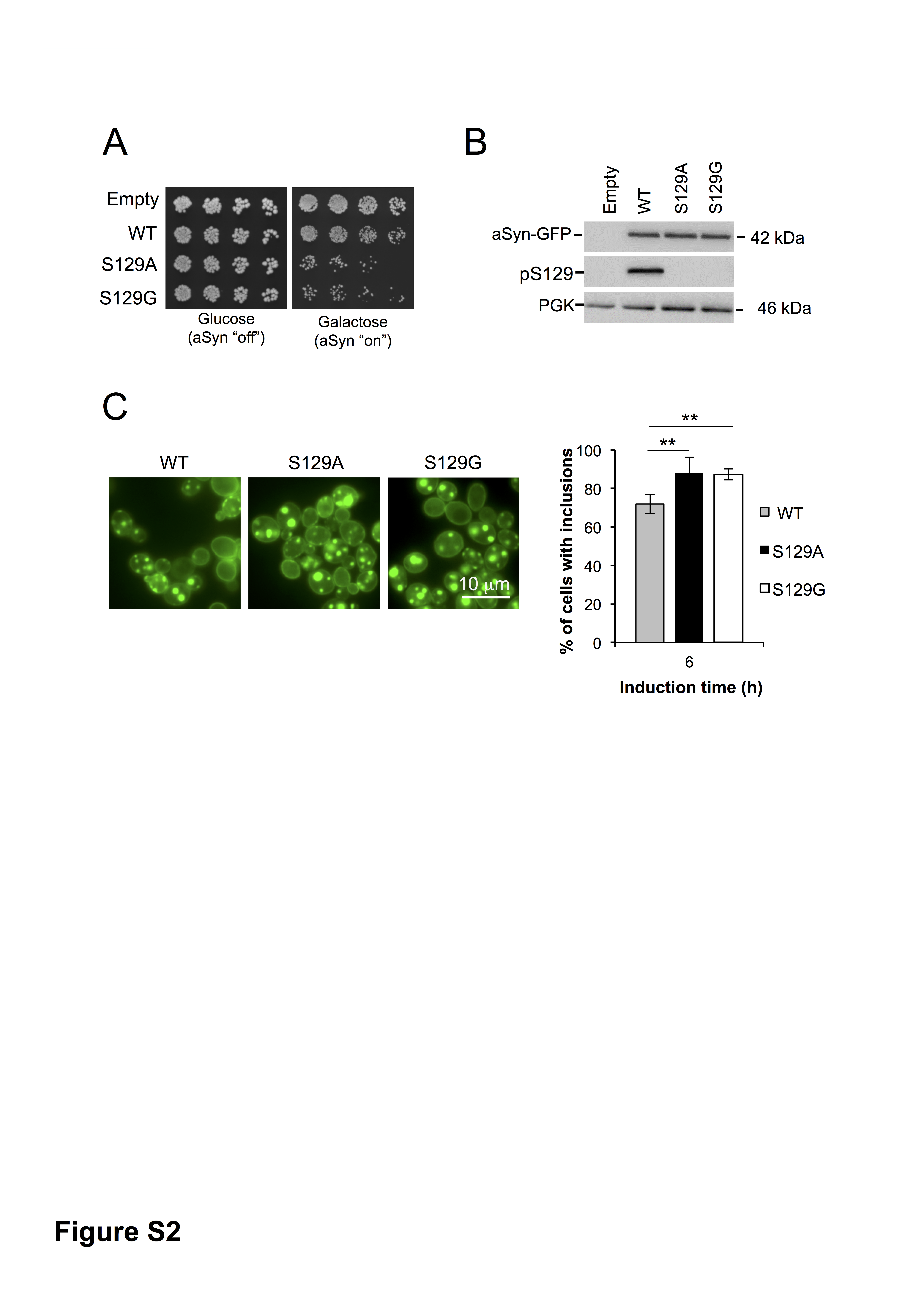

Supplement: Figure S2 — S129G aSyn behaves similarly to S129A asyn. (A) Spotting assay of the indicated yeast cells. The cell suspensions with adjusted OD600nm were serially diluted and spotted onto the surface of solid medium containing either glucose (control) or galactose (induced aSyn expression) as carbon source. Results shown are from one representative experiment from at least three independent experiments. (B) WT, S129A or S129G aSyn-GFP expression and pS129 levels in yeast cells assessed by western blot analysis of total protein extracts 6 hours after aSyn-GFP expression induction. PGK was used as loading control. (C) Intracellular localization of the WT, S129A or S129G aSyn-GFP (left panel) and percentage of yeast cells containing aSyn inclusions (right panel), after 6 hours of aSyn expression induction, assessed by fluorescence microscopy (**p<0.01; one way ANOVA and post-hoc Tukey test). Results shown are from one representative experiment from at least three independent experiments. Values represent the mean ± SD. (TIFF) [file pgen.1004302.s002.tiff]

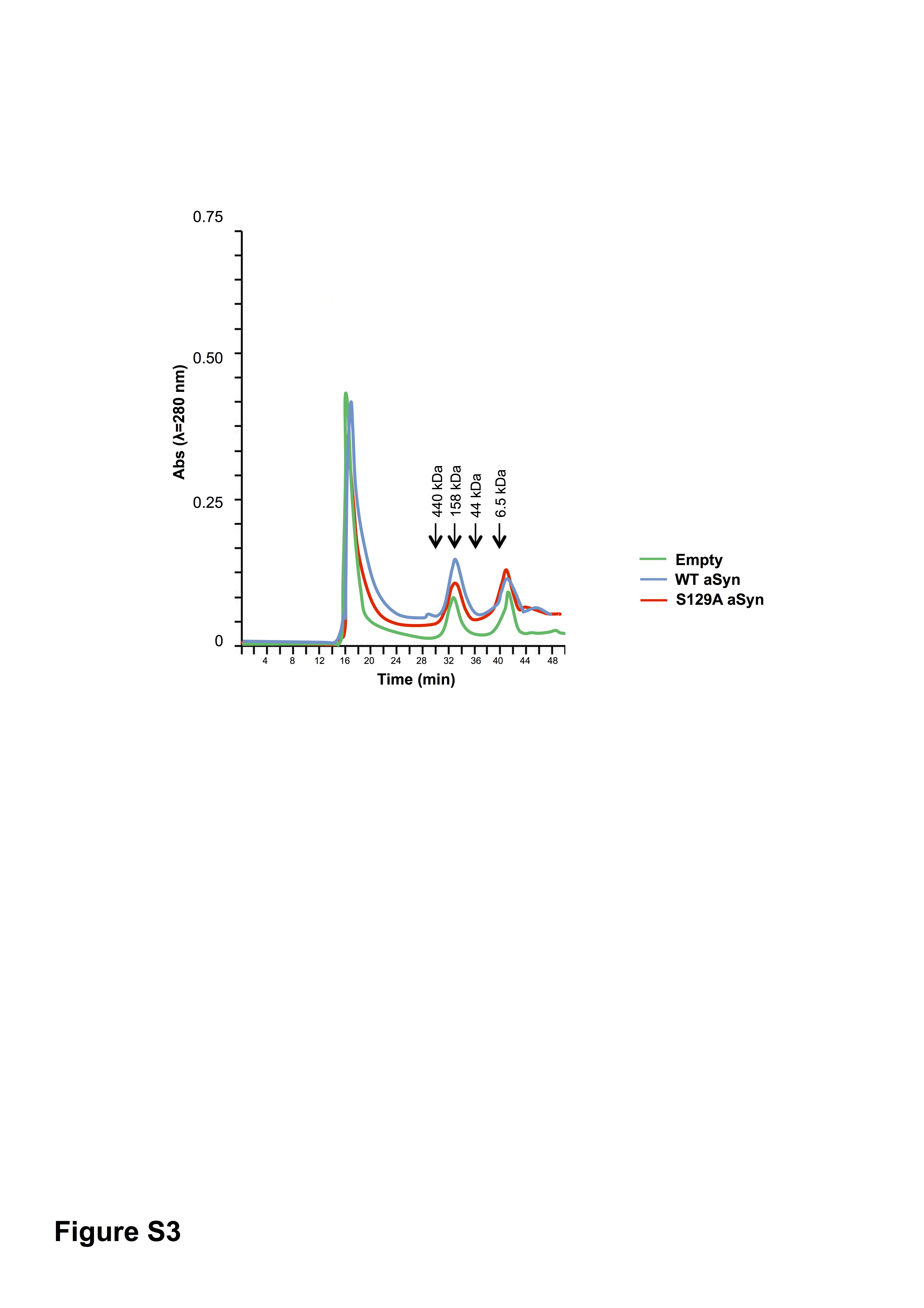

Supplement: Figure S3 — Elution profiles of SEC from total protein extracts of cells expressing WT or S129A aSyn-GFP. Equal amounts of total protein (∼3 mg) from cells not expressing aSyn (empty) or expressing either WT or S129A aSyn-GFP were separated in a Superose 6 10/300 GL column. A calibration curve for SEC was performed using protein molecular weight markers (ferritin, 440 kDa; aldolase, 158 kDa; conalbumin, 75 kDa; ovalbumin, 44 kDa, and aprotinin, 6.5 kDa). The elution time of the protein markers is indicated in the figure. Results shown are from one representative experiment from at least three independent experiments. Values represent the mean ± SD. (TIFF) [file pgen.1004302.s003.tiff]

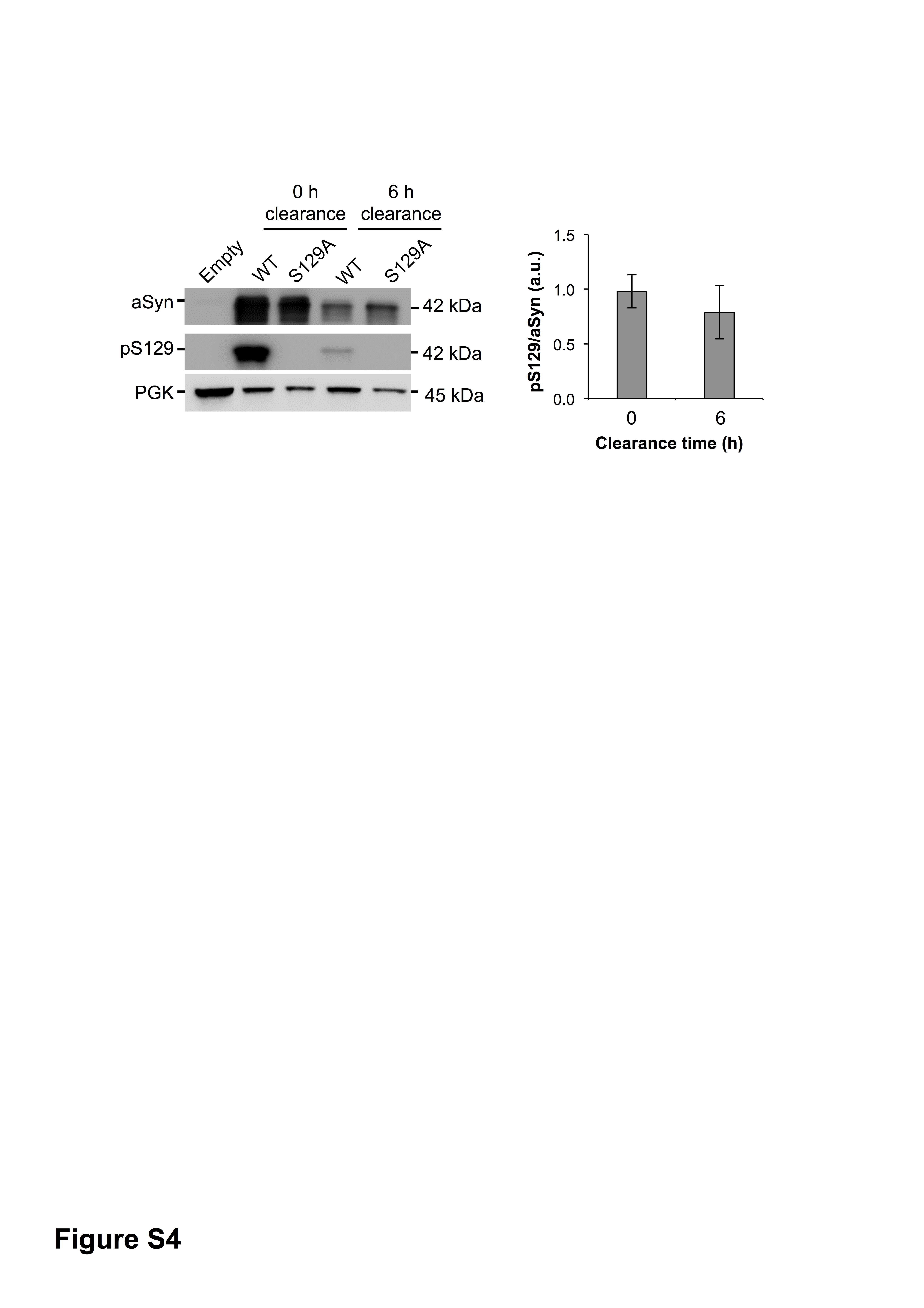

Supplement: Figure S4 — S129 phosphorylation levels of WT aSyn are not altered during aSyn clearance. WT aSyn and pS129 levels at the indicated time points of aSyn clearance (left panel). Densitometric analysis of the pS129-aSyn levels by determining the ratio of the pS129 and the total levels of aSyn (pS129/PGK)/(aSyn/PGK) and normalized to the control. Results shown are from one representative experiment from at least four independent experiments. Values represent the mean ± SD. (TIFF) [file pgen.1004302.s004.tiff]

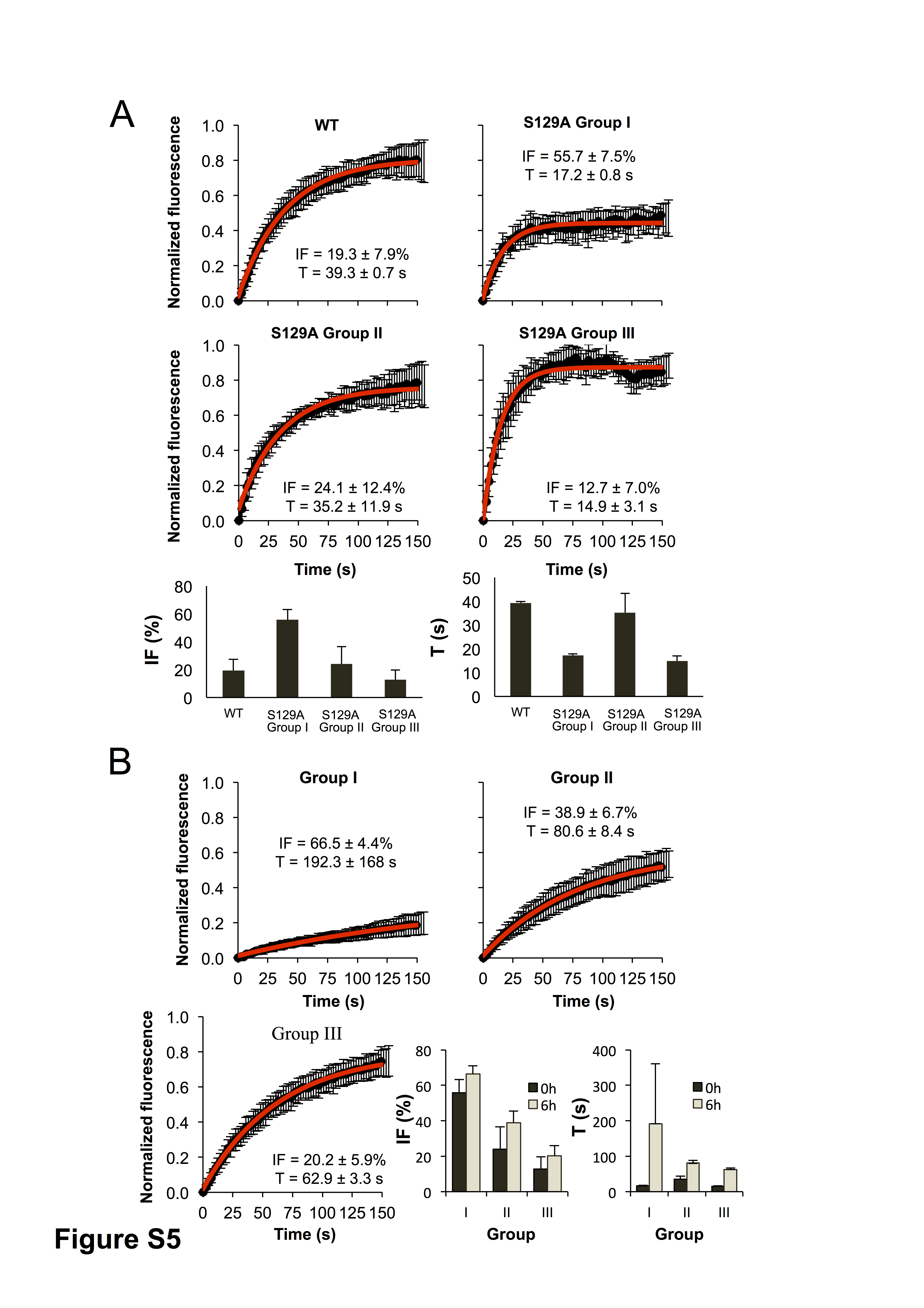

Supplement: Figure S5 — aSyn protein dynamics in inclusions. FRAP recovery curves of the aSyn WT and S129A inclusions shown in Figure 5 are well fit with a single exponential curve. FRAP experiments were performed at (A) 0 hours and (B) at 6 hours of clearance. The aSyn immobile fraction (IF) and the aSyn mean residence time (T) values were calculated from the single exponential fit. Values represent the mean ± SD. (TIFF) [file pgen.1004302.s005.tiff]

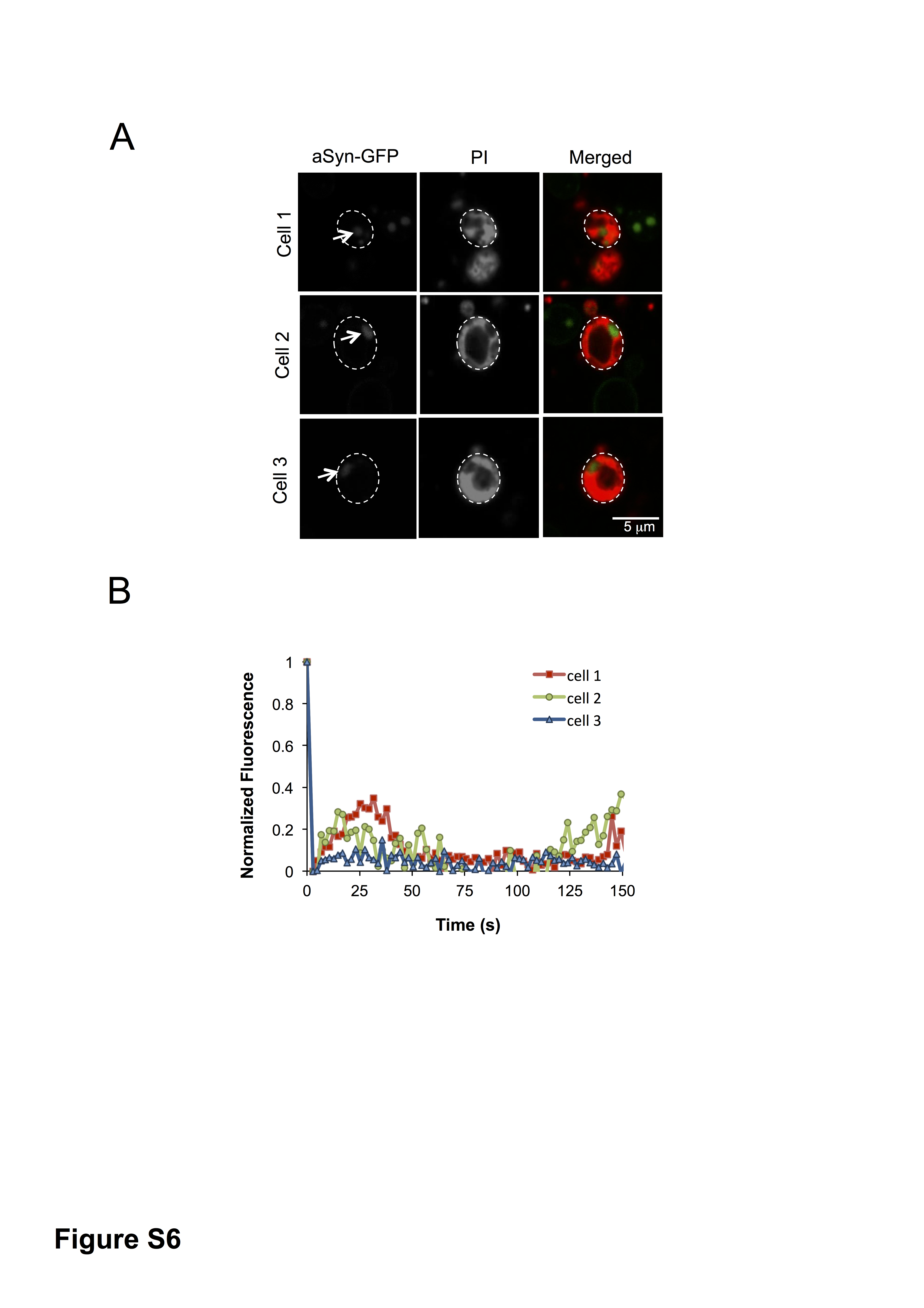

Supplement: Figure S6 — aSyn inclusions from PI-positive cells expressing S129A aSyn do not recover after photobleaching. (A) Time lapse recording of the fluorescence recovery after photobleaching of aSyn inclusions in three representative PI positively marked cells expressing aSyn S129A after 6 hours of expression induction and the corresponding (B) FRAP recovery curve of cells shown in (A). (TIFF) [file pgen.1004302.s006.tiff]

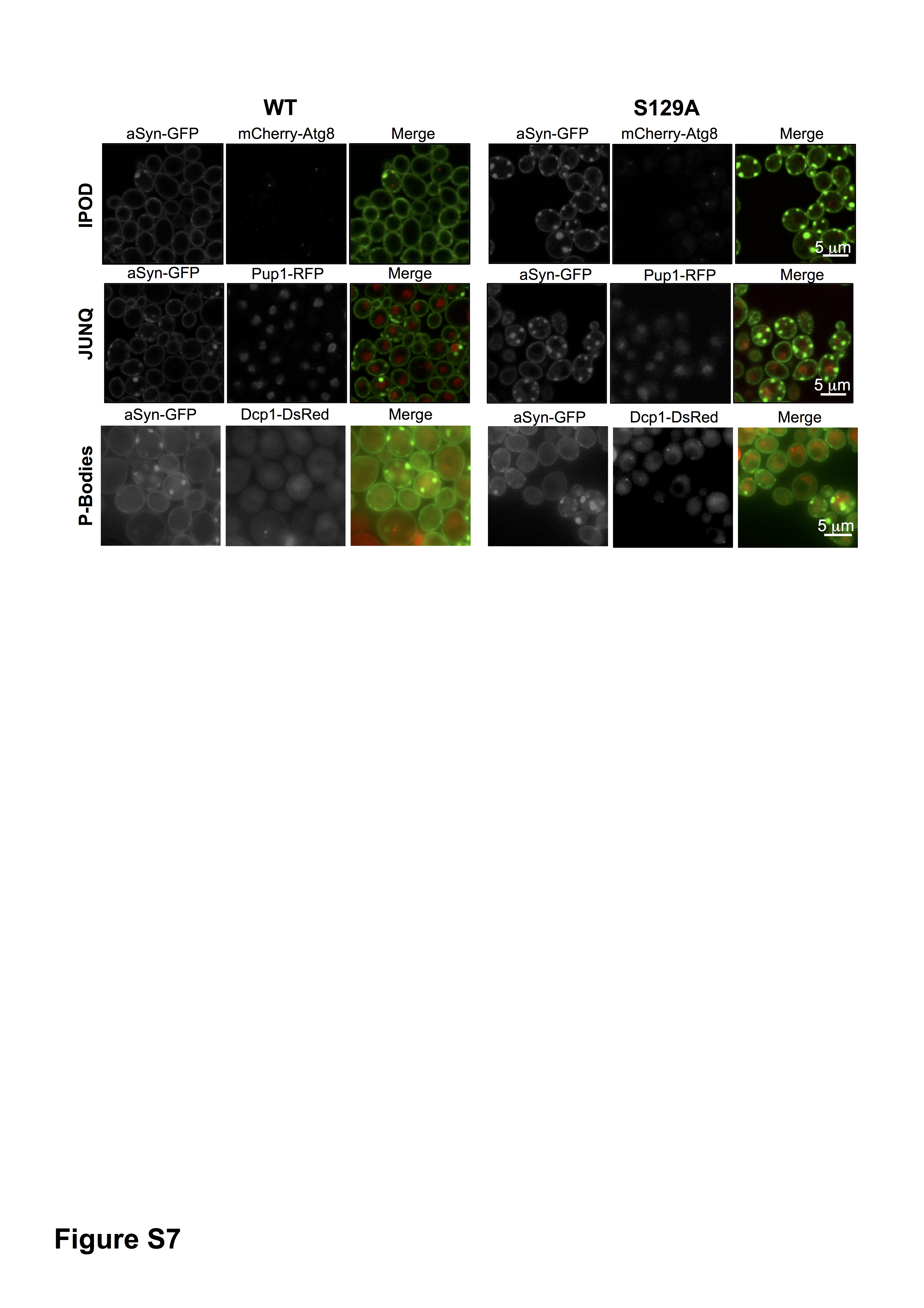

Supplement: Figure S7 — Inclusions formed by WT or S129A aSyn do not colocalize with IPOD, JUNQ or P-bodies markers. Confocal microscopy images of cells co-expressing either WT or S129A aSyn-GFP and mCherry-Atg8, Pup8-RFP or Dcp1-DsRed after 6 hours of aSyn expression induction. Results shown are from one representative experiment from at least three independent experiments. (TIFF) [file pgen.1004302.s007.tiff]

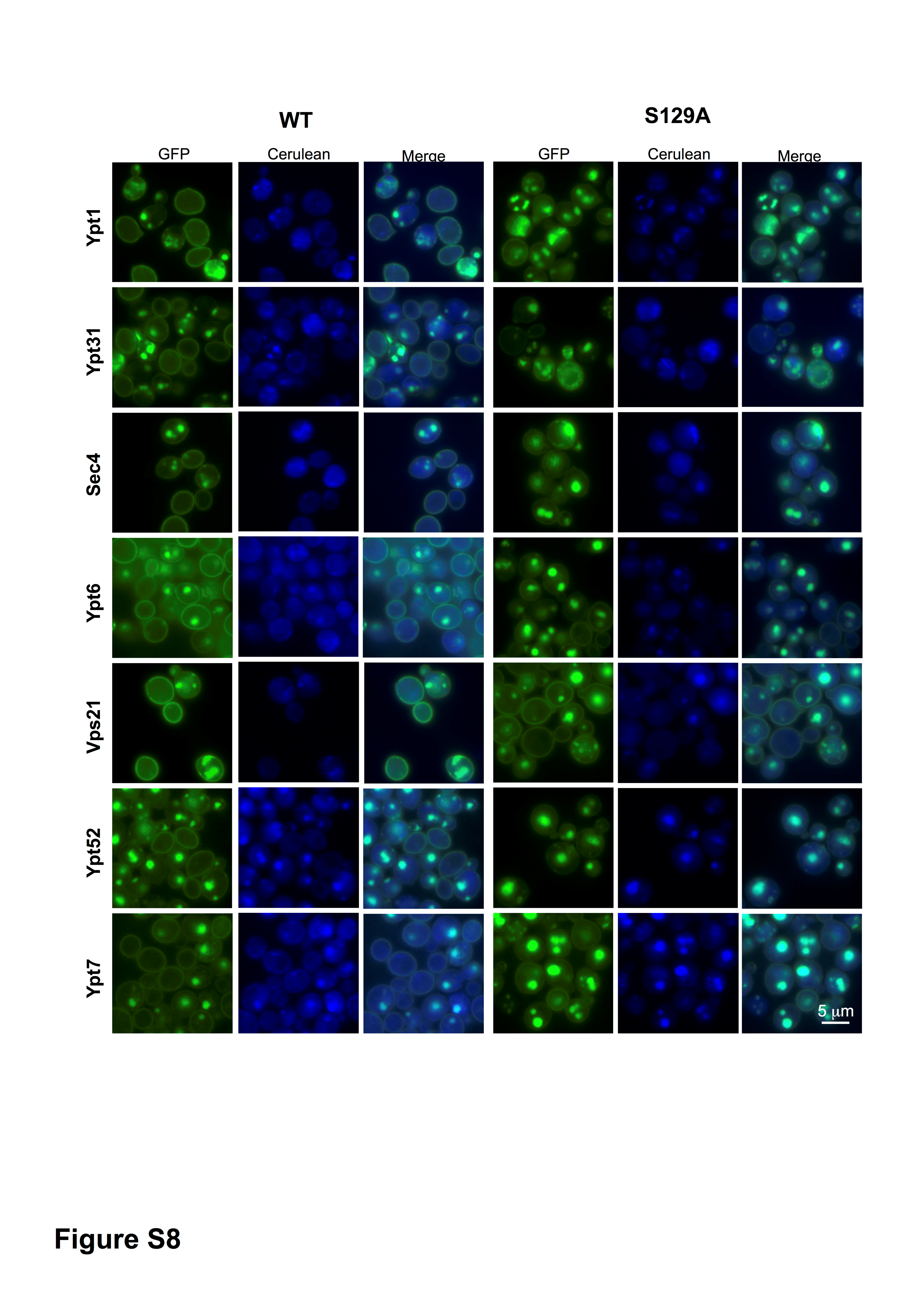

Supplement: Figure S8 — Inclusions formed by WT or S129A aSyn colocalize with vesicular trafficking markers. Fluorescence microscopy images of cells co-expressing either WT or S129A aSyn-GFP, and the indicated vesicular trafficking markers with N-terminal fusions with cerulean, after 6 hours of aSyn expression induction. Results shown are from one representative experiment from at least three independent experiments. (TIFF) [file pgen.1004302.s008.tiff]

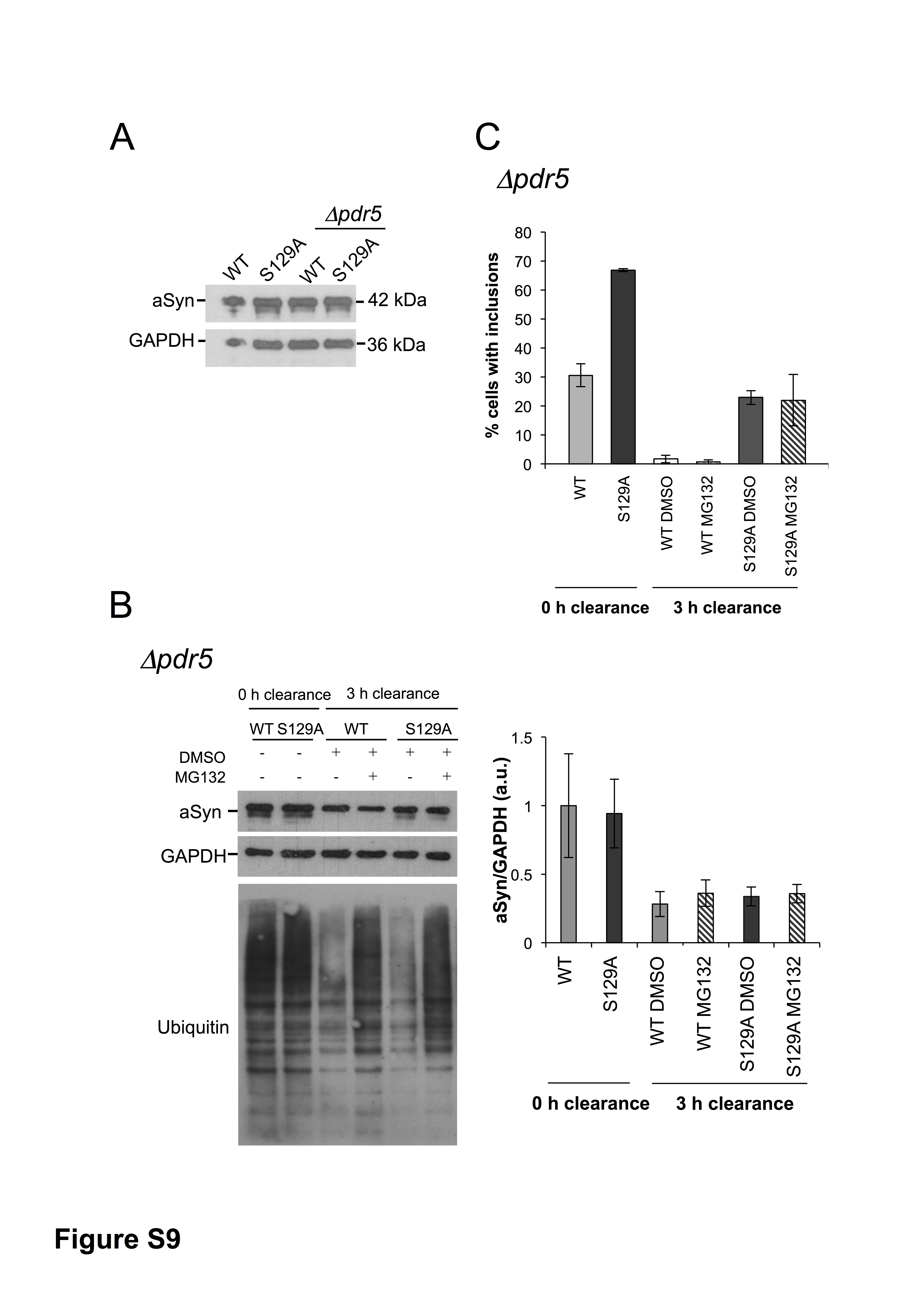

Supplement: Figure S9 — The proteasome is not involved in the clearance of S129A aSyn-GFP. (A) WT, S129A aSyn-GFP expression levels in WT or Δpdr5 yeast cells assessed by western blot analysis of total protein extracts 6 hours after aSyn-GFP expression induction. (B) Percentage of Δpdr5 cells with WT or S129A aSyn-GFP inclusions, before and after 3 hours of clearance in the presence of MG132 in DMSO or only DMSO. (C) WT and S129A aSyn-GFP expression levels at the same time points and subject to the same treatments as in (B), determined by western blot analysis of protein total extracts from Δpdr5 yeast cells. The total levels of ubiquitinated proteins were also evaluated by immunoblotting to confirm the effectiveness of the MG132 as proteasome activity inhibitor. Results shown are from one representative experiment from at least three independent experiments. Values represent the mean ± SD. (TIFF) [file pgen.1004302.s009.tiff]

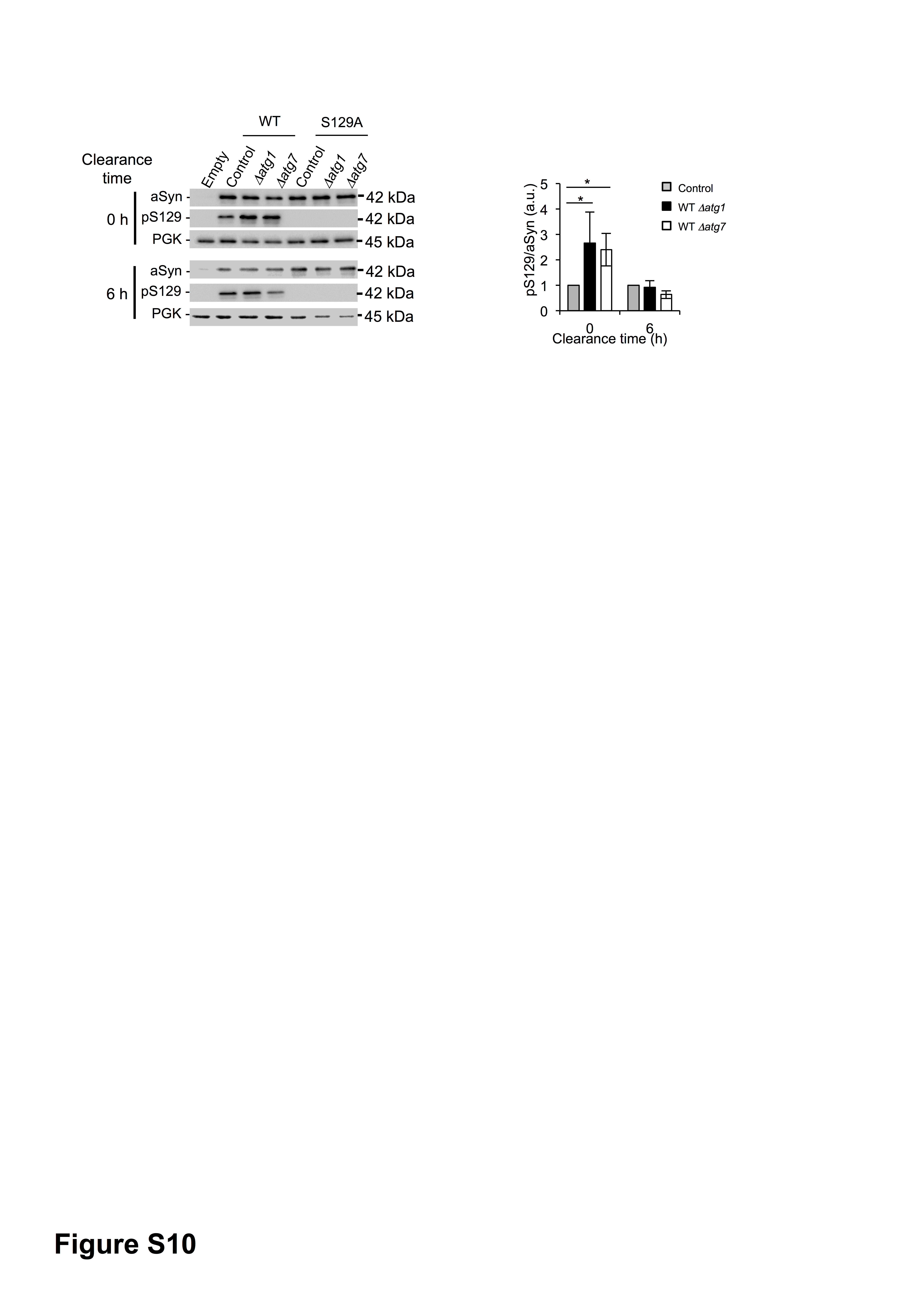

Supplement: Figure S10 — Impairment of autophagy do not affect aSyn S129 phosphorylation. WT aSyn-GFP pS129 levels assessed by western blot analysis of total protein extracts at the indicated time points of aSyn-GFP clearance (left panel). Densitometric analysis of the pS129-aSyn levels by determining the ratio between pS129 and the total levels of aSyn (pS129/PGK)/(aSyn/PGK) and normalized to the control (right panel) (*p<0.05; one way ANOVA with Bonferroni's multiple comparison test). A representative result is shown from at least three independent experiments. Values represent the mean ± SD. (TIFF) [file pgen.1004302.s010.tiff]
